# Memory Updating and Mental Arithmetic

**DOI:** 10.3389/fpsyg.2016.00072

**Published:** 2016-02-02

**Authors:** Cheng-Ching Han, Tsung-Han Yang, Chia-Yuan Lin, Nai-Shing Yen

**Affiliations:** ^1^Department of Psychology, National Chengchi UniversityTaipei, Taiwan; ^2^Research Center for Testing and Assessment, National Academy for Educational ResearchNew Taipei City, Taiwan; ^3^Department of Psychology, University of YorkYork, UK; ^4^Research Center for Mind, Brain, and Learning, National Chengchi UniversityTaipei, Taiwan

**Keywords:** multidigit mental multiplication, updating working memory, domain-general updating ability, domain-specific updating ability, task difficulty

## Abstract

Is domain-general memory updating ability predictive of calculation skills or are such skills better predicted by the capacity for updating specifically numerical information? Here, we used multidigit mental multiplication (MMM) as a measure for calculating skill as this operation requires the accurate maintenance and updating of information in addition to skills needed for arithmetic more generally. In Experiment 1, we found that only individual differences with regard to a task updating numerical information following addition (MUcalc) could predict the performance of MMM, perhaps owing to common elements between the task and MMM. In Experiment 2, new updating tasks were designed to clarify this: a spatial updating task with no numbers, a numerical task with no calculation, and a word task. The results showed that both MUcalc and the spatial task were able to predict the performance of MMM but only with the more difficult problems, while other updating tasks did not predict performance. It is concluded that relevant processes involved in updating the contents of working memory support mental arithmetic in adults.

## Introduction

Numerate societies demand that their members have numerical skills. Most previous studies have focused on simple arithmetic problems to understand how cognitive factors influence the acquisition of mathematical skills. For example, some of these studies have examined the contribution of working memory to simple multiplication fact retrieval problems (e.g., Meyer et al., 2010; Soltanlou et al., 2015). Cognitive neuropsychological studies have shown that left parietal structures play an important role in simple calculation. In particular, the left angular gyrus mediates the retrieval of multiplication facts from memory (Chochon et al., 1999; Lee, 2000; Zamarian et al., 2009), whereas a frontal-parietal network is involved in more complex calculation processes (Lucchelli and De Renzi, 1993; Grabner et al., 2009). These findings suggest that the mechanisms for complex multiplication calculation processes involve both parietal and frontal mechanisms. However, the contribution of working memory to complex multiplication calculation requires additional investigation. In the present study, we focus on multidigit rather than single-digit multiplication because it exemplifies a package of needed skills: understanding the number system and the principles of arithmetic, memory for the retrieval of multiplication facts, procedures for multiplying and adding, and the ability accurately to maintain and update information as needed by the task (e.g., Hunter, 1962; Baddeley and Hitch, 1977; Ericsson and Charness, 1994; Logie et al., 1994; Ericsson and Kintsch, 1995). Competence in this task can be seen as evidence of the possession of important numerical skills.

Which cognitive factors promote the acquisition of calculation skills? Two broad categories have been proposed. First, there is the category of *domain-general* capacities that are held to promote learning across a wide range of topics. These include intelligence or general cognitive capacity (Kovas et al., 2007), spatial ability (Geary et al., 2007), and working memory. Second, *domain-specific* capacities in the domain of numbers are needed (Butterworth, 1996, 2010; Iuculano et al., 2011).

The model of working memory (WM) proposed by Baddeley and Hitch (1977) has multiple components, including a Central Executive (CE), a Phonological Loop (PL), and a Visuo-Spatial Sketchpad (VSSP). PL has been associated with solving single-digit addition (Hecht, 2002), while the VSSP has been linked with the encoding of visually presented problems (Logie et al., 1994). The Central Executive (CE) system has been thought to play a key role in aspects of calculation requiring the storage and manipulation of intermediate results online by updating the results of operations such as carrying and borrowing. In this model, the CE was originally thought of as the system “to which all the complex issues that did not seem to be […] specifically related to the two subsystems were assigned” (Baddeley, 2003). The basic idea of this model, then, is that calculation, along with a wide range of other tasks, makes use of a complex domain-general cognitive system.

Updating processes are intuitively plausible as the principal locus of the WM contribution to calculation, since intermediate results from operations such as carrying and borrowing are required by mental computation, but where CE and PL can be experimentally distinguished, it is CE that seems more critical for calculation. For example, articulatory suppression, which is expected to interfere with PL but not CE, did not affect calculation; while random interval generation, thought to be the responsibility of CE, did reduce arithmetical performance (De Rammelaere et al., 2001). Furthermore, it has been found that children with specific difficulties in arithmetic do not differ on tasks that rely primarily on PL, such as immediate serial recall (digit span), but do perform worse on tasks tapping CE (Siegel and Ryan, 1989; McLean and Hitch, 1999). Further evidence also indicates that children with mathematical difficulties have a CE deficit connected to processing of numerical and visual information (Andersson and Lyxell, 2007). On the other hand, individual differences in PL have been associated with arithmetic impairments (Gathercole et al., 2004). A recent fMRI study also shows that the horizontal segment of the left intraparietal sulcus (IPS) is involved in processing order information in verbal WM, number order judgment, and alphabetical order judgment (Attout et al., 2014).

The most convincing evidence relating WM to mathematical achievement in children comes from longitudinal studies. Geary et al. (2009) deployed the Working Memory Test Battery for Children (Pickering and Gathercole, 2001) to measure CE, PL, and VSSP separately, from kindergarten to third grade. They found that different WM systems are able to discriminate between different levels of mathematical impairments in children, as well as different levels of mathematical proficiency. Specifically, Geary and colleagues underline the importance of the CE component for the ability of correctly retrieving simple addition facts.

Similarly, in a longitudinal study by De Smedt et al. (2009a), the CE was a unique predictor of both first- and second-grade attainment in mathematics. They used two complex span tasks, namely counting span (Case et al., 1982) and listening span (Daneman and Carpenter, 1980), as measures of CE function, instead of an updating task. Interestingly, there were age-related differences in the role of components of WM, such that the visuo-spatial sketchpad was a unique predictor of first-grade, but not second-grade, mathematics attainment, whereas the phonological loop emerged as a unique predictor of second-grade, but not first-grade, mathematics achievement. However, Soltanlou et al. (2015) found that the phonological loop emerged as a predictor of third-grade multiplication performance, whereas visuo-spatial WM was a predictor of fourth-grade performance. Meyer et al. (2010) also showed that CE and the phonological loop promote performance in mathematical learning in early stages, whereas visuo-spatial WM is more important in later stages. It seems that although CE or updating ability in general plays an important role in predicting math performance, more specific capacity may also mediate performance.

Iuculano et al. (2011) found that the ability to update a CE function differentiated typically from in 9-year olds with low attainment scores on arithmetic. However, they found that it was updating specifically numerical information that was predictive, not updating equivalent non-numerical information. In the present study, we would like to assess whether domain-general memory updating ability or the capacity for updating specifically numerical information is better able to predict calculating skills in a multidigit multiplication task. Specifically, we want to test this idea further in three main ways:

1. Test whether individual differences in calculation ability in adults still depends on updating WM. Here we use multidigit mental multiplication because it requires accurate maintenance and updating of information, as well as an extensive package of skills needed for arithmetic more generally, namely understanding the number system and the principles of arithmetic, the memory of multiplication facts and their retrieval, and procedures for multiplying and adding.
2. Iuculano et al. (2011) used two tasks. One required updating numerical information: in this case, the 1, 2, or 3 largest numbers from a spoken sequence. The other required updating non-numerical size information: the 1, 2, or 3 largest animals in a spoken sequence. Here we will use tasks that require updating different types of information. The objective is to determine whether individual differences in calculation ability depend on individual differences in the ability to update in general, or, instead, the ability to update a specific type of information. Here we test three potentially separable updating processes: updating verbal information, updating numerical information, and updating spatial information.
3. We will assess whether the contribution of updating to calculation ability is specific to more difficult, or to all multidigit mental multiplication problems.

## Experiment 1

In this experiment, we assessed the contribution to multidigit mental multiplication (MMM) ability of three different memory abilities, measured as follows: one involved numbers and updating memory as the result of simple addition and subtraction; another required memory for letters but no updating; and the third required memory of spatial locations but no updating. These three complex WM tasks are taken from the battery developed by Lewandowsky et al. (2010). This design allows us to assess both the domain-general WM factor and also the domain-specific factors contributed by each of the processing tasks, namely numbers, verbal material processing, and spatial memory.

### Materials and methods

#### Participants

There were 48 participants in the study. The average age was 24, the standard deviation was 3.23; half were males and half females. Most were students at the National Chengchi University, Taiwan. The study was approved by the Regional Ethics Committee at National Taiwan University. All participants gave informed consent before taking part in the experiment. The data of only 47 subjects were included in the analysis; one participant was excluded for his accuracy rate in the mental multiplication task, which was below average by three standard deviations.

#### Experimental procedure

There were four tasks in this experiment: a multidigit mental multiplication task, the dependent measure, and three working memory tasks. Half of the participants performed these tasks in the following sequence: multidigit mental multiplication task followed by three working memory tasks. The other half was given the reversed sequence: the three working memory tasks followed by the multidigit mental multiplication task.

#### Multidigit mental multiplication task (MMM)

##### Stimuli

There were four types of problems used in the experiment. They were: 2 digits multiplied by 1 digit (2 × 1), 3 digits multiplied by 1 digit (3 × 1), 4 digits multiplied by 1 digit (4 × 1), and 2 digits multiplied by 2 digits (2 × 2). Examples for each type of multiplication problems were: 2 digits multiplied by 1 digit (35 × 4), 3 digits multiplied by 1 digit (356 × 4), 4 digits multiplied by 1 digit (3567 × 4), and 2 digits multiplied by 2 digits (35 × 67).

According to cognitive load theory (Chandler and Sweller, 1991; Tuovinen and Sweller, 1999), the presence of too many elements for a human to process in visual or verbal working memory can cause overloading. We hypothesized that, if a multiplication problem requires additional steps to be calculated, WM loading may increase, which results in impaired calculating performance. For example, in the problem “43x2 = ?” presented in an elementary school math class, a teacher would teach you to multiply the digits in the ones column first, then to multiply the digits in tens, and so on as the digits increase. In the simplest situation, where there is no number to be carried, problems of the type “2 digits multiplied by 1 digit” (2 × 1) include two steps: multiply the ones and then the tens by the one digit. The type “3 digits multiplied by 1 digit” (3 × 1) includes three steps, namely multiply the ones, tens, and hundreds by one digit.

In the current study, we initially defined the problem difficulty by the number of the calculating steps. We selected four types of multiplication problems, namely type 2 × 1, 3 × 1, 4 × 1, and 2 × 2. Types 2 × 1, 3 × 1, and 4 × 1, respectively, include at least two, three, or four calculation steps. Type 2 × 2 includes the steps of multiplying the ones and tens by the first digit, and then multiplying the ones and tens by the second digit. Finally, it is necessary to sum them. Five steps at least are required.

There were 80 trials in the task. The trials consisted of four types of multiplication problems and 20 trials for each condition. Twenty trials of each type of problem were randomly selected from a multiplication problems pool (see Appendix 1 in Supplementary Material) for each participant. The digits forming the multiplication problems in this task were restricted to the digits 2–9. Both 1 and 0 were excluded in multiplicands and multipliers. The digit 5 was excluded in multipliers. The digits used in the multiplicands and multipliers were constrained to numbers without repeated digits.

##### Procedure of task

**Figure 1:**
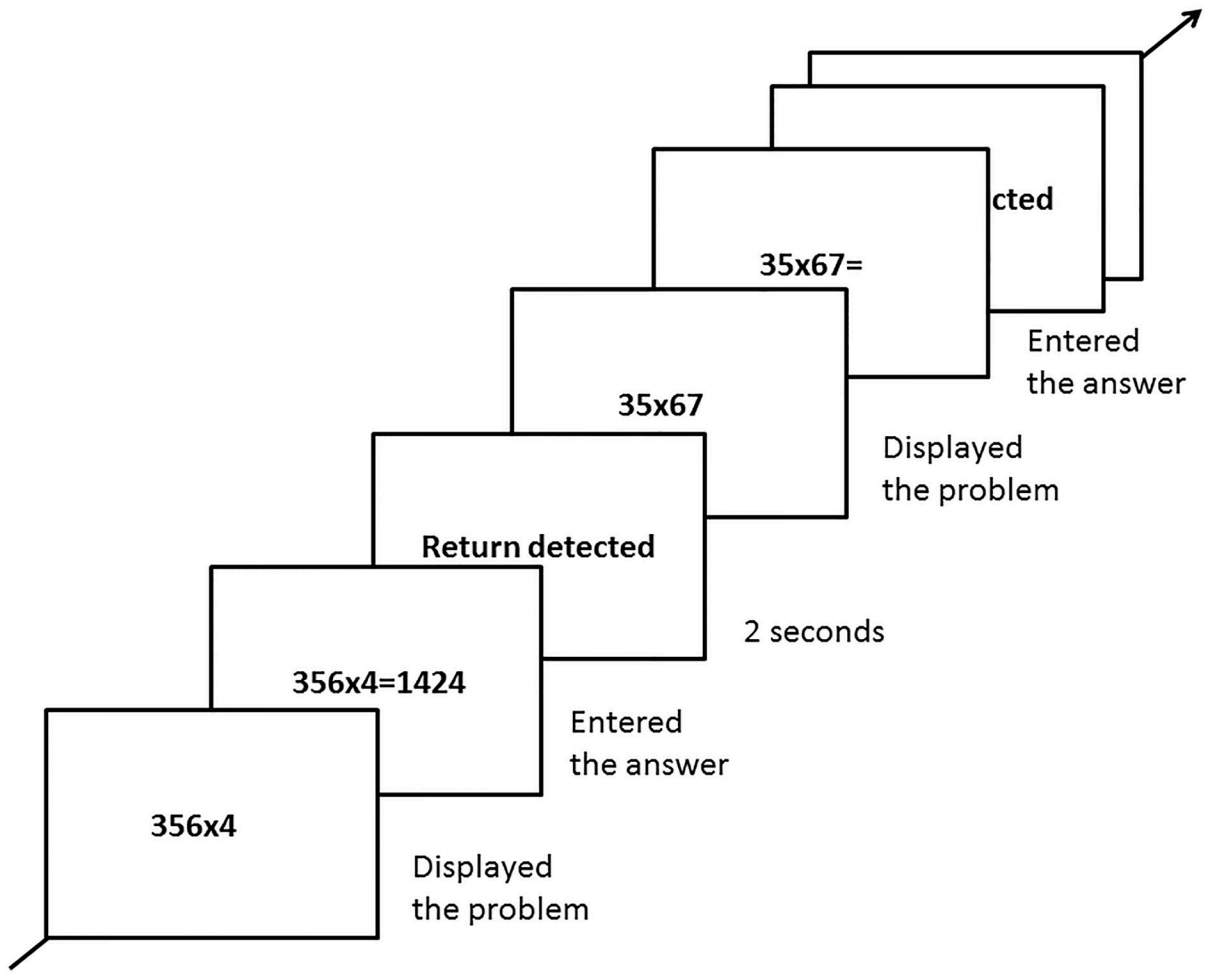
**Experimental procedure (e.g., the 3 × 1 and 2 × 2 problem types showing in center of screen) for the multidigit mental multiplication(MMM) task**.

At the beginning of each trial, one multiplication problem (2x1, 3x1, 4x1, or 2x2) was displayed at the center of the screen. Participants were required to calculate it mentally, and use the number pad (showing the digits 0–9) to enter the answer as quickly and as correctly as possible. There was no time limit set for making responses. The participant's answer appeared on the screen after entering. After sending the answer, by pressing the enter button, they could press the enter button again to go on to next trial. A screen with the sentence “return detected” in the center for 2 s appeared between each trial (See Figure 1). A total of 80 trials was presented in random sequence, and the participants had one short break not to exceed 1 min after completing each set of 20 trials, until the end of this task. In this task, we collected accuracy and reaction time (RT) as dependent variables from participants.

#### Memory tasks

**Figure 2:**
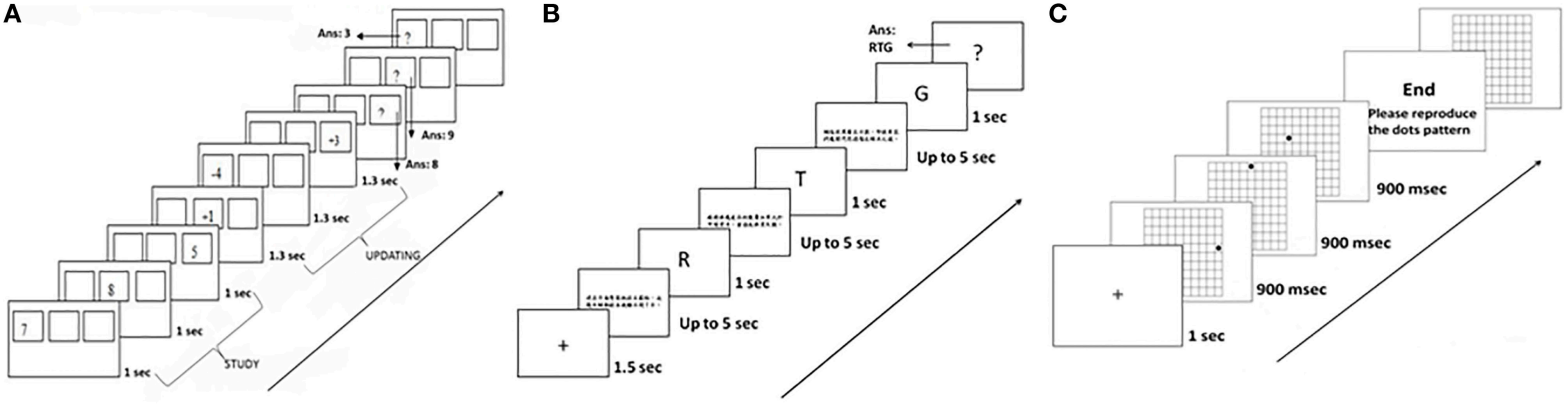
**The experimental procedures for the three working memory tasks in Experiment 1: (A) MUcalc: calculation updating task, (B) Mletter: letter memory task, and (C) Mspace: spatial memory task**.

We adapted the working memory tasks from the battery developed by Lewandowsky et al. (2010), as follows (See Figure 2).

##### Calculation updating task (MUcalc)

In each trial of this task, 3–5 rectangular frames were simultaneously shown on the screen. During the study period, a single digit appeared in a frame (e.g., 7 or 8) for 1 s. The participants had to remember the digit for that particular frame. During the updating period, an arithmetic operation (e.g., −4 or +1) showed in a particular frame. Participants had 1.3 s to remember the updated result at that moment in that frame (e.g., 8+1 = 9). The number of updating operations varied from two to six in each trial. This updating process continued until a question mark “?” appeared in a frame. This was the recall period, in which participants were required to enter the memorized updated results into each frame. Thus, what the participants had to do in this memory-updating task was to update and remember the last result computed for each frame. There was no limitation on response time, but the answer could not be changed after being entered. After entering all digits, the next trial began. Before the test trials, the participants did two practice trials to familiarize themselves with the task. There were 15 experimental trials in the task (See Figure 2A). We collected task accuracy as a dependent measure.

##### Letter memory task (Mletter)

The structure of this task is based on the “reading span task” (Daneman and Carpenter, 1980). Here, participants had to update their memory with a sequence of letters in the context of judging whether a sentence presented on a computer screen made sense. In the beginning of a trial, a cross at the center of screen was shown for 1.5 s. Then a sentence was presented for up to 5 s, for participants to make a judgment. Thereafter, a letter appeared on the screen for 1 s, for participants to memorize. This sequence was repeated three to seven times for each trial. At the end of a trial, participants had to recall the letters in the presented order when a question mark “?” appeared on the screen. The response time for recall was unlimited, but the answer could not be changed after being entered. After entering all letters, the next trial proceeded. Before the test trials, participants did three practice trials to familiarize themselves with the task (See Figure 2B). This task comprised 15 trials. Notice that this task does not directly involve updating. Rather, the task requires adding to memory rather than modifying elements already in it. The accuracy we collected from task was the dependent measure.

##### Spatial memory task (Mspace)

Here, the participants saw a grid of cells (10 × 10) on the screen. For each trial, a cross at the center of screen was shown for 1 s, and then a black dot was shown in a cell for 900 ms. Dots were shown in different locations of cells in the grid during the experiment. The participants were asked to remember the relative location of the cells in which the dots appeared on each trial (2–6 dots). After all dots had been displayed, “End—Please reproduce the dot pattern” was shown on the screen. The participants could replace the pattern of dots in an empty grid on the screen by using the mouse to click on the location of cells. The participants could delete a dot by clicking on it again. The answer would be considered correct if the spatial relations between the dots were reproduced correctly. The response time for recall was unlimited; after recalling the number and relative position of dots in that trial, a “Next” word button appeared on the screen. The participants could click on this “Next” button when they were satisfied with their response. There were two practice trials before the test trials; the task comprised 30 trials (See Figure 2C). Notice that this task does not directly involve updating. The participant simply had to remember the location of the dots. That is, the task requires adding to memory rather than modifying elements already in it. Similarly to the last two memory tasks, the accuracy of the task was the dependent measure.

## Results

### Correlations between multidigit mental multiplication performance (MMM) and the memory tasks

Our main purpose was to investigate how updating working memory contributes to multidigit mental multiplication (MMM); thus it was decided to perform a correlation test at the beginning.

**Figure 3:**
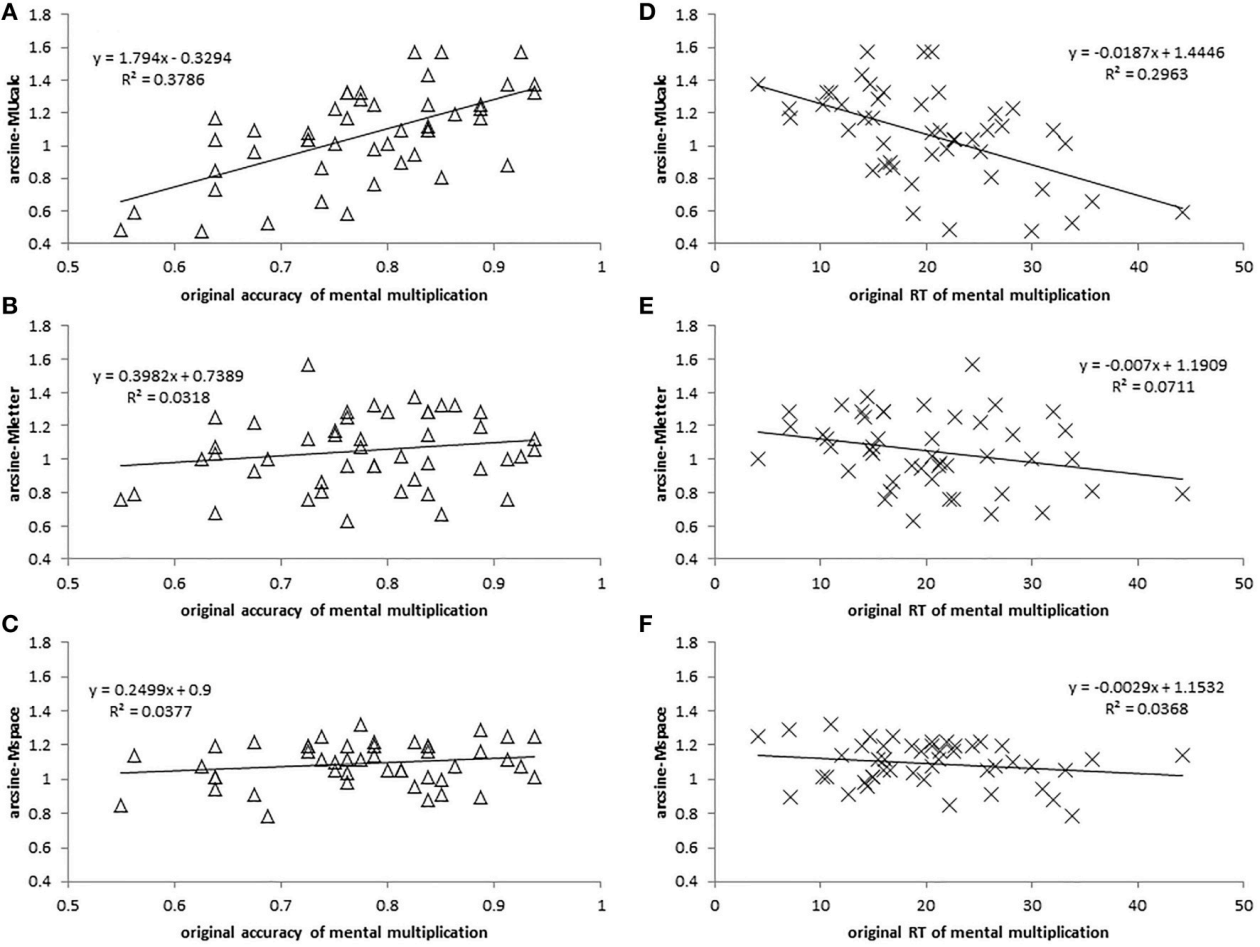
**Correlation plots for Experiment 1**. Correlations are respectively as depicted: **(A)** between arcsine-accuracy of MUcalc and original accuracy of MMM, **(B)** between arcsine-accuracy of Mletter and original accuracy of MMM, **(C)** between arcsine-accuracy of Mspace and original accuracy of MMM, **(D)** between arcsine-accuracy of MUcalc and original RT of MMM, **(E)** between arcsine-accuracy of Mletter and original RT of MMM, and **(F)** between arcsine-accuracy of Mspace and original RT of MMM.

In order to meet the normality assumption of the Pearson correlation test, we examined the data distributions of all tasks. The data were arcsine-transformed if the distribution of any tasks failed to reach normality. Finally, the data from three updating tasks (MUcalc, Mletter, Mspace) were arcsine-transformed according to the Shapiro-Wilk criterion (owing to our N = 47 < 50). Finally, we used both the original accuracy and the RT of MMM and arcsine-transformed accuracies of three updating WM to perform the Pearson correlation test (See Table 1 and Figure 3).

**Table 1:** **Experiment 1. Correlations between original accuracies and RTs of multidigit mental multiplication (MMM) task with the arcsine accuracies of three memory tasks (MUcalc, Mletter, and Mspace)**.

***N* = 47**	**Multidigit mental multiplication task**		**Working memory tasks**		
	**Accuracy**	**RT**	**MUcalc**	**Mletter**	**Mspace**
**MULTIDIGIT MENTAL MULTIPLICATION TASK**					
Accuracy	1	−0.462^**^	0.615^***^	0.178	0.194
RT		1	−0.544^***^	−0.267	−0.192
**MEMORY TASKS**					
MUcalc			1	0.574^***^	0.247
Mletter				1	0.092
Mspace					1

For correlation between accuracy and RT of multidigit mental multiplication (MMM) task: ^**^ p < 0.01.

For correlations between original accuracy and RT of multidigit mental multiplication (MMM) task and the arcsine-accuracies of three memory tasks, p levels that are significant after Bonferroni correction for multiple comparisons: ^***^p < 0.000167 (0.001/6).

For correlations between the arcsine-accuracies of three memory tasks, p levels that are significant after Bonferroni correction for multiple comparisons: ^***^p < 0.00033 (0.001/3).

Zero-order individual correlations between tasks in the study after Bonferroni correction are presented in Table 1. The results showed that MUcalc was highly correlated with MMM in terms of accuracy and RT. The accuracy of MUcalc was highly and positively correlated with the accuracy of MMM (*r* = 0.615; 95% confidence interval: 0.379–0.852; *p* = 0.000004), and was negatively correlated with RT of MMM (*r* = −0.544; 95% confidence interval: −0.796 to −0.292; *p* = 0.000076). Therefore, better MUcalc means faster and more accurate MMM. However, the accuracy of neither Mletter nor Mspace was significantly correlated with any indices of MMM after the Bonferroni correction. In addition, with regard to working memory tasks, the tasks MUcalc and Mletter were correlated with each other (*r* = 0.574; 95% confidence interval: 0.328–0.819; *p* = 0.000025; See Table 1).

Multiple regression analysis further confirmed the relationship between tasks of working memory and those of MMM. We used three updating working memory tasks (arcsine scores) to predict the original accuracy and RT of MMM. On accuracy, only MUcalc (*Beta* = 0.755, *p* < 0.001) significantly [*F*_(3, 43)_ = 10.596, *p* < 0.001] predicted and accounted for 38.5% of the variance on MMM performance (adjusted *R*^2^ = 0.385, standard error of the estimate = 0.076). On RT, the MUcalc (*Beta* = −0.566, *p* = 0.001) significantly [*F*_(3, 43)_ = 6.218, *p* = 0.001] predicted and accounted for 25.4% of the variance on MMM performance (adjusted *R*^2^ = 0.254, standard error of the estimate = 7.103).

### MMM, item difficulty, and memory updating

To examine how participants with different MMM accuracies performed on different problem types, we used the total MMM score (the average score of all types: 2 × 1, 3 × 1, 4 × 1, and 2 × 2) as each participant's performance index, and divided total 47 participants into three performance groups according to this index. The low performance group had 15 participants (*M* = 0.665, *SD* = 0.060), the medium had 16 (*M* = 0.784, *SD* = 0.025), and the high had 16 (*M* = 0.877, *SD* = 0.038). The mean and standard error for each condition is showed in Table 2.

**Table 2:** **Experiment 1. The means and standard errors (SEs) of multidigit mental multiplication (MMM) accuracies arranged by four types of problems and those of working memory accuracies arranged by three types of tasks in different MMM performance groups**.

***N* = 47**	**Types of multidigit mental multiplication tasks**				**Types of working memory tasks**		
**Accuracy**	**2x1**	**3x1**	**4x1**	**2x2**	**MUcalc**	**Mletter**	**Mspace**
**TOTAL**							
Mean	0.917	0.861	0.727	0.596	0.842	0.847	0.882
SE	0.009	0.011	0.016	0.016	0.022	0.016	0.009
**HIGH PERFORMANCE GROUP**							
Mean	0.963	0.919	0.850	0.778	0.924	0.861	0.881
SE	0.016	0.019	0.027	0.028	0.020	0.027	0.016
**MEDIUM PERFORMANCE GROUP**							
Mean	0.925	0.878	0.725	0.606	0.871	0.864	0.894
SE	0.016	0.019	0.027	0.028	0.030	0.026	0.010
**LOW PERFORMANCE GROUP**							
Mean	0.863	0.787	0.607	0.403	0.725	0.814	0.870
SE	0.016	0.019	0.028	0.029	0.043	0.029	0.019

A 3 multiplication performance groups (a between variable with three levels: low, medium, high) × 4 problem types (a within variable with four levels: 2 × 1, 3 × 1, 4 × 1, 2 × 2) two-way mixed ANOVA revealed that both group performance and problem type achieved significant main effects, *F*_(2, 44)_ = 93.980, *F*_(3, 132)_ = 112.285, both *p*s < 0.001, $\eta_{\text{p}}^{2}$ = 0.810 and 0.718 respectively. In group performance, the high scoring group did better on MMM than the medium and low score groups, and the medium score group did better than the low score group (all *p*s ≤ 0.001). In problem types, the participants had the best accuracy percentages in 2x1 problems, but the worst in 2x2 problems (all *p*s ≤ 0.001). That is, the easier the problem type, the higher the accuracy. This was consistent with our prediction that performance would decline as the number of calculation steps increased.

A significant interaction, *F*_(6, 132)_ = 7.061, *p* < 0.001, $\eta_{\text{p}}^{2}$ = 0.243 showed that, in problems of the types 2x1 and 3x1, participants of the medium score group performed as well as the high score group, and both groups performed better than the low score group (all *p*s ≤ 0.025). However, when calculating type 4x1 and 2x2 problems, significant discrimination revealed that the high score group still did the best, followed by the medium group, and the last was, as expected, the low score group (all *p*s ≤ 0.011). Therefore, differences in MMM performance were revealed by the different problem types. High-performing students could do well at all types of the MMM problems. As for medium performance students, the interaction showed that they did equally well in both type 2x1 (*M* = 0.925, *SD* = 0.058) and 3x1 (*M* = 0.878, *SD* = 0.071) problems, but worsened significantly when calculating 4x1 (*M* = 0.725, *SD* = 0.075) and 2 × 2 (*M* = 0.606, *SD* = 0.115) problems. Type 4x1 problems may be a critical boundary for our university student samples.

We also carried out a one-way between-subject ANOVA to confirm whether the three groups would differ on the updating WM tasks. As expected, in the MUcalc task [*F*_(2, 44)_ = 10.315, *p* < 0.001, $\eta_{\text{p}}^{2}$ = 0.305], the result showed that participants who were in the high or medium multiplication group performed better than participants who were in the low multiplication group (*p*s ≤ 0.007). No significant main effects were revealed in both Mletter and Mspace tasks. Notice that all the WM tasks were performed to a similar level. Here are the means standard errors of accuracy in MUcalc, Mletter, and Mspace were 0.842 (0.022), 0.847 (0.016), and 0.882 (0.009), respectively (See Table 2).

### Discussion of experiment 1

Overall, MMM and the memory tasks showed good ranges of individual differences. MUcalc was correlated with Mletter (*r* = 0.574) but not Mspace, suggesting that both MUcalc and Mletter may draw upon a common set of cognitive resources, but Mspace appears to share little of the common resources with these two tasks. However, each task still drew upon some different cognitive resources for representing different stimulus content and processes.

Our immediate objective was to assess which of the tasks, if any, tapped competences that contributed to MMM. MUcalc was significantly correlated with MMM accuracy (*r* = 0.615) and speed (*r* = -0.544). But neither Mletter nor Mspace were significantly correlated with MMM accuracy and speed. Further ANOVA analysis showed that participants with different MMM performance did show different accuracies in each problem type. For example, in the easier type 2 × 1 and 3 × 1 problems, medium and high scoring group participants performed at the same level (Medium group: *M*_2*x*1_ = 0.925 and *M*_3*x*1_ = 0.878; High group: *M*_2*x*1_ = 0.963 and *M*_3*x*1_ = 0.919), but the medium score participants were significantly worse than the high score group in calculating type 4x1 problems (Medium group: *M* = 0.725; High group: *M* = 0.850). That is, type 4x1 was harder for medium performance participants in our student sample. For the low score group participants, their performances became worse as the calculating steps of problem type increased; the mean range was from 0.863 (type 2 × 1) to 0.403 (type 2 × 2), whereas the mean range was from 0.963 (type 2 × 1) to 0.778 (type 2 × 2) for high scoring participants.

MUcalc, unlike Mletter and Mspace, involves both numbers and calculation, albeit simple single-digit addition and subtraction, and it may have been the case that the observed relationships with MMM were due to these common elements rather than to a number-specific updating process. In MUcalc, elements to be remembered were required to be changed as a result of addition or subtraction: that is, the updating altered elements held in memory. By contrast, Mletter and Mspace involved updating only by adding to memory rather than modifying elements already in it. Therefore, we designed three new tasks for Experiment 2, in which there was updating that required altering memorized elements. The elements of two of these tasks did not involve numbers, while those of one did. Further, we wished to see whether it was the mere presence of numbers or the requirement to carry out calculations on numbers that predicted MMM success.

## Experiment 2

In order to clarify whether the significant correlations between MUcalc and both the accuracy and RT of multidigit mental multiplication (MMM) stems from the mere presence of numbers or the requirement to carry out calculations on numbers, three modified memory updating tasks were developed for Experiment 2.

1. A *spatial memory-updating* task (MUSpace) requires participants to update the location of a dot moving clockwise or counterclockwise. No numbers or arithmetic calculations are involved in the spatial memory-updating task.
2. A *numerical memory-updating* task (MUNumber) requires participants to update and remember the larger number of two numbers, with no arithmetic calculation involved.
3. A *word memory-updating* task (MUWord) requires participants to update and remember the smaller animal denoted by two animal words (similar to a task used in Iuculano et al., 2011). For example, in their task, participants were asked to remember the three smallest animals “pelican (
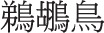
), tortoise (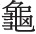), and chicken (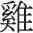)” from an animal list presenting six items “giraffe (
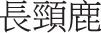
), pelican (
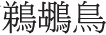
), tortoise (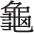), tiger (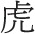), chicken (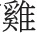), dolphin (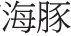).”

By comparing the correlations between different memory updating tasks and MMM, we expected to be able to clarify whether the significant correlation between original MUcalc and MMM are due to updating domain-specific information, or to domain-general updating capacity. In the MMM task, we selected only two problem types from Experiment 1: type 2 × 1 and type 4 × 1. The former was generally easy for all groups of college students, who performed at a level of at least 91.8% accuracy. The 4x1 type was more difficult, as was shown by medium level students in Experiment 1. In type 2x1 and 3x1 problems, medium level students calculated as well as high-level students. By choosing type 2x1 and type 4x1 problems, it was possible to assess the effects of the MMM difficulty in relation to updating capacity.

### Materials and methods

#### Participants

Fifty-eight participants completed all the tasks. Most of them were students at National Chengchi University, Taiwan. The study was approved by the Regional Ethics Committee at National Taiwan University. All participants gave informed consent before taking part in the experiment. Five of them were excluded from further analyses because of low performance on at least one task (below average by more than 3 standard deviations); 53 subjects (17 males and 36 females; average age: 21.72, *SD* = 2.78) were thus analyzed in this study.

#### Multidigit mental multiplication (MMM) task

The MMM task in this experiment was similar to the MMM task used in Experiment 1, but with only two problem types from Experiment 1 (2 × 1 and 4 × 1), comprising 50 questions of each type, thus making 100 questions in total. Fifty questions of each type of problems were randomly selected from a multiplication problems pool (see Appendix 2 in Supplementary Material) for each participant. The questions were pseudo-randomly assigned into four blocks. Participants were instructed to take a short rest (around 1 min) if needed between blocks. In each trial, a fixation with 2 or 4 s was followed by a multiplication question displayed in the center of the screen. Participants were instructed to answer correctly as soon as possible, and were told that the time limit of each question was 50 s. We used a different response procedure for this task from that used in Experiment 1 to double check the results in Experiment 1. Instead of entering the answers directly with number keys, participants were instructed to complete each question by pressing only four buttons in the current task, namely, “1,” “2,” “3,” and “4.” The function of button “1” was to increase numbers, button “2” was to decrease numbers, button “3” shifted places, and button “4” output the answer. For example, a question was displayed as “24 × 3 = 00”; since the answer is 72 in this example, and the order to respond was always from left to right (i.e., from tens to units), participants had to press the button “1” seven times (or the button“2” three times) to adjust the first number from 0 to 7. After the adjustment of tens was completed, participants pressed the button “3” to change the place from tens to units, and then adjust the units by pressing the button “1” twice (or the button “2” eight times). Last, participants pressed the button “4” to indicate that the question was answered (the place being adjusted was displayed with a gray background so that participants could easily tell which place they were modifying). The questions remained on the screen until participants gave an answer or the time was up. If an answer was given in time, the string “Answer confirmed” would display for 2 s on the screen before the fixation of next trial began; otherwise, a string “Too slow” would appear instead, to remind participants to answer questions within the time limit. In the MMM task, we still collected accuracy and RT as dependent measures. The RT was defined by the interval from the onset of stimulus to participant's first key press response.

#### Memory updating task

**Figure 4:**
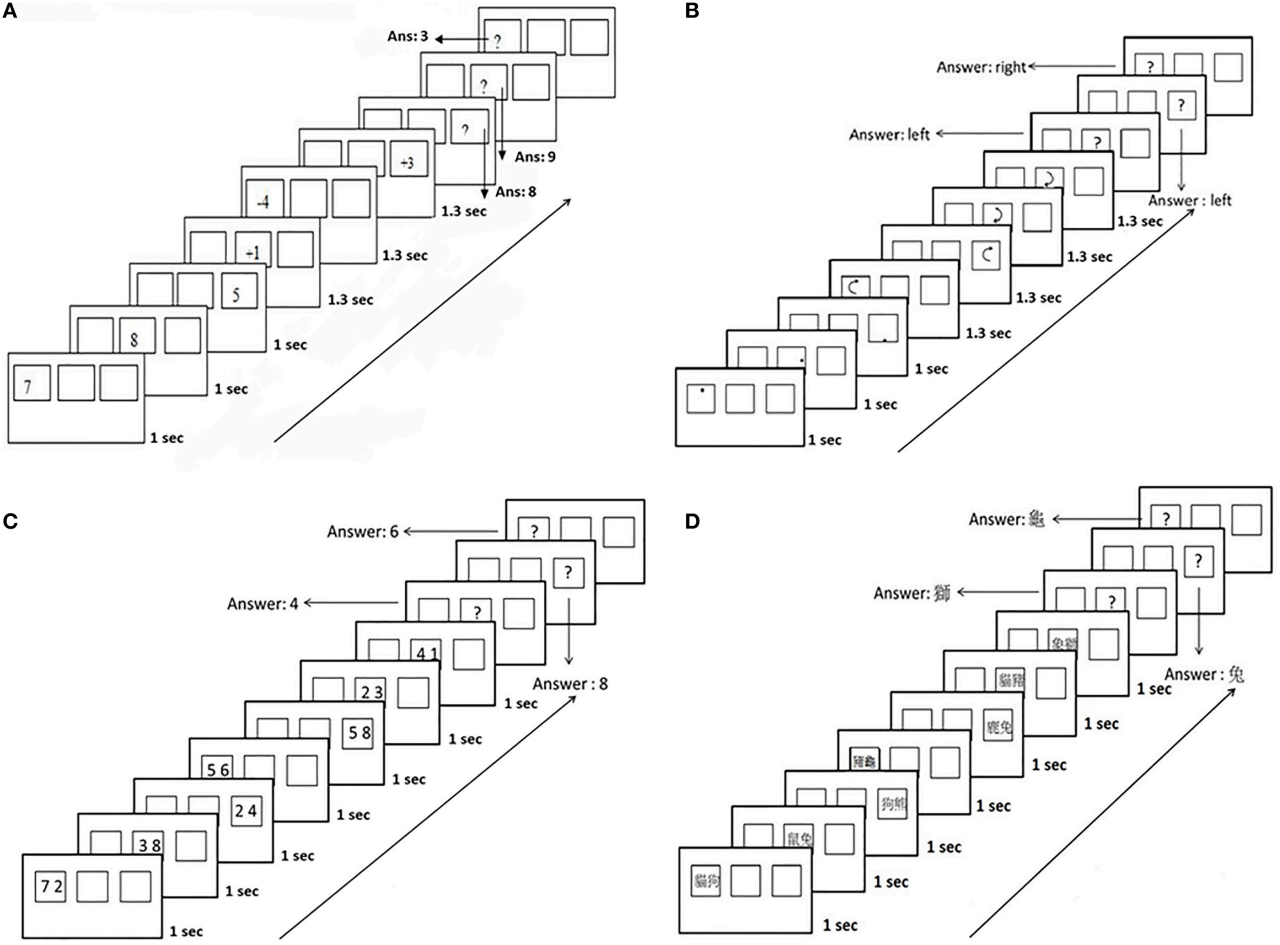
**The experimental procedures of original calculation updating task, MUcalc, and three modified memory updating tasks in Experiment 2: (A) the original memory updating task (MUcalc), (B) the spatial updating task (MUSpace), (C) the numerical updating task (MUNumber), and (D) the word updating task (MUWord)**.

Three new modified memory-updating tasks were developed to explore the role of number memory: a spatial memory updating task (MUSpace), a numerical memory updating task (MUNumber), and a word memory updating task (MUWord) (See Figure 4). There were 15 trials in each task.

##### MUcalc

This was exactly the same task as in Experiment 1: participants were instructed to remember the digit in each rectangular frame, do the arithmetic asked for, and give the final result of each rectangular frame separately at the end of each trial. The accuracy of MUcalc task was still the dependent measure.

##### MUSpace

This task was modified from the MUcalc task, but used dots and clockwise/counter-clock wise marks instead of digits and addition/subtraction. In each trial, 3–5 rectangular frames were shown on the screen (just as in MUcalc). During the study period, one dot appeared in a frame, and the dot was in one of four possible locations, i.e., left, right, top, or bottom, of the frame, for 1 s. Participants had to remember the location of the dot in a particular frame. During the updating period, a directional mark, either clockwise or counterclockwise, was shown in a particular frame for 1.3 s. On the occurrence of a clockwise mark in the frame, participants had to update the dot location from its previous location in their memory to a location 90 degree clockwise: for example, from “top” to “right.” If the counterclockwise mark was shown, participants were to mentally move the dot location counterclockwise, for example, from “top” to “left.” The number of updating operations varied from 2 to 6 times in a trial (the same as in MUcalc). This process continued until a question mark “?” appeared in the frame. This was the recall period, when participants had to enter the memorized updated results into each frame by using the number pad. The number “8” on the number pad represents the “top location,” “6” on the number pad represents the “right location,” “2” represents “low,” and “4” represents the “left location.” In addition, the order for the results were pseudorandomized (participants were not always given answers from left to right or from right to left). Thus, what participants had to do in this spatial memory-updating task was to update and remember the last location of the dot in each frame. There was no time limit for the response, but the answer could not be changed after responding. After entering all digits, the next trial proceeded. Before the test trials, the participants did two practice trials to familiarize themselves with the task. There were 15 trials in this task (See Figure 4B). The accuracy of the task was the dependent measure.

##### MUNumber

This task was also modified from the MUcalc task, but participants were required to remember the updated digits without doing arithmetic. The number of rectangular frames and the operations were the same as the tasks described above. During the study period, two digits appeared side by side at the same time on a frame for 1 s, e.g., 7 and 2. Participants had to remember the larger digit in a particular frame (7, in this example). During the updating period, another two digits were displayed (e.g., 3 and 8) in a particular frame. Participants had to update the larger digit showing on the most recent frame (8, in this example). This process continued until a question mark “?” appeared in that frame. In this recall period, participants had to enter memorized updated results into each frame with a pseudorandomized order. Thus, what the participants had to do in this numerical memory-updating task was to update and remember the last result for each frame. No time limit was given for the response, but the answer could not be changed after responding. After entering all digits, the next trial proceeded. The number of test trials and the practice trials were the same as other tasks (See Figure 4C). The accuracy of the task was the dependent measure.

##### MUWord

This task was similar to the MUNumber task, but participants were required to remember updated characters representing animals. The number of rectangular frames and the operations were the same as the tasks described above. During the study period, two animal names appeared side by side at the same time in a frame, e.g., 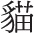 (cat) and 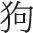 (dog), for 1 s. Participants had to remember the smaller animal denoted by the word in a particular frame (cat in this example). During the updating period, another two animal words were displayed, e.g., 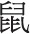 (mouse) and 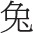 (rabbit), for a particular frame. Participants had to update the smaller animal shown in the most recent frame. This process continued until a question mark “?” appeared on that frame. Participants were instructed to enter the updated results they memorized for each frame with a pseudorandomized order by pressing response keys (the responding keys for each animal were A, S, D, F, G, H, J, K, and L respectively, and the keys and the corresponding animal characters were always displayed as pairs on the screen in this recall stage). Thus, what the participants had to do in this word memory-updating task was to update and remember the last result for each frame. No time limit was given for the response, but the answer could not be changed after responding. After entering all the words, next trial proceeded. The number of test trials and the practice trials were the same as the other tasks (See Figure 4D). Accuracy was collected as dependent variable.

The order of the nine animal characters used in the task, from small to large, were 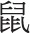 (mouse), 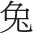 (rabbit), 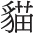 (cat), 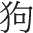 (dog), 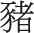 (pig), 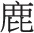 (deer), 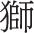 (lion), 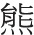 (bear), and 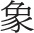 (elephant). This size order was ranked by the other 12 participants in advance. Horse (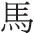), ox (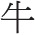), and sheep (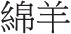) were discarded in this pre-test as participants were confused about their size rankings. This size ranking was given to the participants attending the experiment before the MUWord task began, and the task only began after participants confirmed that they fully understood and remembered the ranking.

### Results

#### Correlations between multidigit mental multiplication performance (MMM) and memory updating ability

**Figure 5:**
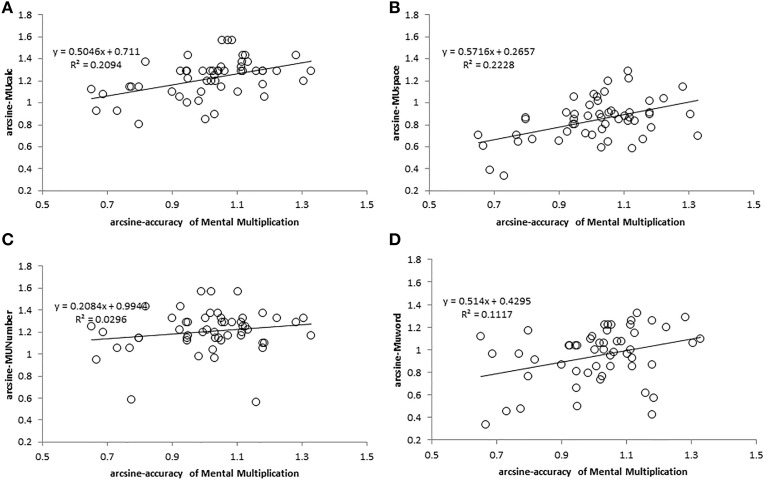
**Correlation plots for accuracies in Experiment 2**. Correlations are respectively as depicted: **(A)** between arcsine-accuracy of MUcalc and arcsine-accuracy of MMM, **(B)** between arcsine-accuracy of MUSpace and arcsine-accuracy of MMM, **(C)** between arcsine-accuracy of MUNumber and arcsine-accuracy of MMM, **(D)** between arcsine-accuracy of MUWord and arcsine-accuracy of MMM.

Again, for investigating the correlation between MMM and memory updating ability, we tested the normality of each task according to Kolomogorov–Smirnov (owing to N = 53 > 50), and transformed the non-normal distribution data by arcsine-transformation. However, the three updating tasks were approximately normal, though not completely. The *p* values were: MUcalc (*p* < 0.001), MUNumber (*p* = 0.035), and MUWord (*p* = 0.048). We reported the correlation between the four arcsine-accuracy of MU tasks and both the arcsine-accuracy and original RT of MMM, as seen in Table 3 and Figure 5.

**Table 3:** **Experiment 2. Correlations between both arcsine-accuracies and original RT of multidigit mental multiplication (MMM) tasks and arcsine-accuracies of four MU tasks (MUcalc, MUSpace, MUNumber, and MUWord)**.

***N* = 53**	**Memory updating tasks**			
	**MUcalc**	**MUSpace**	**MUNumber**	**MUWord**
**MULTIDIGIT MENTAL MULTIPLICATION TASKS**				
**Accuracy**				
All	0.458^*^	0.472^**^	0.172	0.334
2x1	0.189	0.304	0.022	0.062
4x1	0.499^**^	0.422^*^	0.238	0.402
**RT**				
All	−0.115	0.005	−0.136	−0.136
2x1	−0.235	−0.090	−0.078	−0.150
4x1	−0.067	0.036	−0.144	−0.120
**MEMORY UPDATING TASKS**				
MUcalc	1	0.432^**^	0.199	0.389^*^
MUSpace		1	0.346	0.364^*^
MUNumber			1	0.470^*^
MUWord				1

All p levels that are significant after Bonferroni correction for multiple comparisons are marked as follows:

For correlations between both the arcsine-accuracies and original RT of multidigit mental multiplication (MMM) tasks and the arcsine-accuracies of four MU tasks:

^*^p < 0.002083(0.05/24); ^**^p < 0.000416 (0.01/24).

For correlations between arcsine-accuracies of four MU tasks:

^*^p < 0.00833(0.05/6); ^**^p < 0.00167(0.01/6).

In this experiment, the arcsine-accuracy of MMM correlated with both MUcalc (*r* = 0.458; 95% confidence interval: 0.208–0.708; *p* = 0.000571) and MUSpace (*r* = 0.472; 95% confidence interval: 0.224–0.720; *p* = 0.000359) after Bonferroni correction, especially with difficult type 4x1 (MUcalc: *r* = 0.499; 95% confidence interval: 0.256–0.743; *p* = 0.000142; MUSpace: *r* = 0.422; 95% confidence interval: 0.168–0.677; *p* = 0.001632), whereas the RT of MMM did not correlate with any MU tasks. Accuracy of the MUNumber task, which involved comparing digits but with no calculation, was not significantly correlated with the performance of MMM task. MUNumber was also not significantly correlated with either MUcalc or MUSpace (See Table 3). This is reasonable, given that MUNumber was not correlated with MMM.

In addition, MUNumber and MUWord were correlated with each other (*r* = 0.470; 95% confidence interval: 0.222–0.718; *p* = 0.000381), which may result from similar task design and the memory updating process (See Table 3). Multiple regression analysis further confirmed the relationship between memory updating and MMM. We used the four arcsine-accuracy measures of MU tasks to predict the arcsine-accuracy of MMM. The result showed that both MUSpace (*Beta* = 0.323, *p* = 0.025) and MUcalc (*Beta* = 0.277, *p* = 0.051) significantly predicted MMM accuracy [*F*_(4, 48)_ = 5.509, *p* = 0.001] and accounted for 25.8% of the variance (adjusted *R*^2^ = 0.258, standard error of the estimate = 0.135).

#### MMM, item difficulty, and memory updating

The means and standard deviations of original accuracy of the four working memory updating tasks, MUcalc, MUSpace, MUNumber, and MUWord, were 0.926 (0.065), 0.735 (0.126), 0.918 (0.088), and 0.792 (0.155*)* respectively. One-way repeated measures ANOVA showed the significant difference between tasks [*F*_(3, 156)_ = 55.260, *p* < 0.001, $\eta_{\text{p}}^{2}=0.515$]. The performances of MUcalc and MUNumber were significantly better than that of MUSpace and MUWord (both *p*s < 0.001).

Again, in order to examine the different MMM accuracies that participants showed in different problem types, we used the total MMM score (the average score of type 2x1 and 4x1) as each participant's performance index, and divided them into three similar performance groups. The low performance group had 18 participants (*M* = 0.742, *SD* = 0.075), the medium 18 (*M* = 0.859, *SD* = 0.013), and the high 17 (*M* = 0.920, *SD* = 0.025). The mean and standard error for each condition is seen in Table 4.

**Table 4:** **Experiment 2. The means and standard errors (SEs) of multidigit mental multiplication (MMM) accuracies arranged by two types of problems, and those of memory updating accuracies arranged by four types of tasks in different multiplication performance groups**.

***N* = 53**	**Types of multidigit mental multiplication tasks**		**Types of memory updating tasks**			
**Accuracy**	**2x1**	**4x1**	**MUcalc**	**MUSpace**	**MUNumber**	**MUWord**
**TOTAL**						
Mean	0.914	0.767	0.926	0.735	0.918	0.792
SE	0.007	0.012	0.009	0.017	0.012	0.021
**HIGH PERFORMANCE GROUP**						
Mean	0.963	0.876	0.955	0.775	0.918	0.816
SE	0.013	0.021	0.008	0.027	0.026	0.040
**MEDIUM PERFORMANCE GROUP**						
Mean	0.916	0.802	0.933	0.772	0.939	0.845
SE	0.013	0.021	0.016	0.024	0.011	0.019
**LOW PERFORMANCE GROUP**						
Mean	0.861	0.622	0.893	0.660	0.898	0.716
SE	0.013	0.021	0.017	0.032	0.024	0.042

A 3 multiplication performance groups (a between variable with three levels: low, medium, high) × 2 problem types (a within variable with two levels: 2x1, 4x1), two-way mixed ANOVA revealed that group performance, problem type, and their interactions showed significant effects, *F*_(2, 50)_ = 65.814, *F*_(1, 50)_ = 92.059, and *F*_(2, 50)_ = 9.411 respectively, all *p*s < 0.001, $\eta_{\text{2}}^{2\mathrm{pt}}$p = 0.725, 0.648, and 0.273 respectively. In group performance, the high scoring group still performed better on MMM than medium and low score groups, and the medium score group performed better than the low score group (all *p*s = 0.001). In problem types, as in Experiment 1, participants showed better accuracy in the type 2x1 problems than in the type 4x1 problems. In the interactions, low performance group participants got worse performance as the problem difficulty increased. Under type 2x1 condition, the *p*-value between low and medium group was 0.011, whereas the *p*-value was lower than 0.001 between these two groups under type 4x1 condition. Obviously, for low scoring participants, their performance diminished greatly as the difficulty of MMM increased.

We again performed a one way between-subject ANOVA to examine whether participants with different MMM performance differed in accuracy on the 4 MU tasks. The results showed that on both MUcalc and MUSpace tasks [*F*_(2, 50)_ = 4.744 and 5.681, *p* = 0.013 and 0.006, $\eta_{\text{p}}^{2}=0.0159$ and 0.185 respectively], participants who were in the high performance group in the MMM task also performed better than participants who were in the low performance group (both *p*s = 0.017). In MUWord, although it was not correlated with MMM, there was shown a significant main effect of group [*F*_(2, 50)_ = 3.751, *p* = 0.030, $\eta_{\text{2}}^{2\mathrm{pt}}$p = 0.130]. The Bonferroni comparisons showed that participants who were in the medium score group in the MMM task performed better than participants who were in the low score group (*p* = 0.035). There was no difference between the groups on the MUNumber task.

#### Discussion of experiment 2

The results of Experiment 2 revealed that both MUSpace and MUcalc were the best predictors for the accuracy of the multidigit mental multiplication task (MMM), not only correlated with MMM, but also with harder (higher load) type 4x1 problems. No numbers or arithmetic calculations are involved in the spatial memory-updating task. On the other hand, the numerical memory-updating task, MUNumber, which involved numbers but neither arithmetic nor spatial operations, showed no significant correlation with MMM problems in this experiment. The results suggested that significant correlations between MUcalc and the accuracy of MMM observed in Experiment 1 were due to updating involved in the calculation process rather than the mere involvement of numbers^1^.

In addition, the RT of MMM was not correlated with any of the MU tasks, contrary to our findings in Experiment 1. It was possible that participants' RTs in Experiment 2 were influenced by means entering responses using only four response buttons, which may not reveal the “real” reaction time precisely^2^.

## General discussion

These updating tasks clearly show that the processes involved in updating the contents of working memory are critical to multidigit mental multiplication (MMM) in adults. In Experiment 2, we replicated the findings in Experiment 1, showing that memory updating involves calculation, MUcalc, predicted performance on the more difficult MMM items (type 4x1), which requires more steps (load) to calculate.

On the other hand, the numerical memory-updating task, which involved numbers but not calculation, was not correlated with performance in MMM problems. This contrasts with Iuculano et al. (2011), who found that individual differences in arithmetic ability was indeed related to updating numbers in a task that required successive comparison of numerical magnitudes (as in MUNumber) but not calculation. However, there are important differences between their study and ours. Their dependent arithmetical ability task was performance on the achievement subscale of the *Dyscalculia Screener* (Butterworth, 2003) which measured accuracy and speed of single-digit addition in terms of norms for an age-group. Moreover, they tested children aged eight- to nine-years old, whereas we tested college students with a mean age of 24 years. It is possible that the contribution of different components or aspects of WM depend on the stage of arithmetical development (as was found by De Smedt et al., 2009b; Meyer et al., 2010; Soltanlou et al., 2015). So, for example, for the children in the Iuculano et al. study, being good at remembering numbers is important for fluent calculation, and individual differences in this ability will therefore play a part in differentiating calculation ability. In our study, MUNumber was strongly correlated with MUWord, suggesting that numbers were being maintained and updated in their phonological form rather than in a form relevant to calculation. Another possible difference is that the percentage correct in MUNumber in our study is quite high. The reason we did not find a correlation between MUNumber and MMM may be the ceiling effect. This possibility could be tested in the future.

By contrast, the spatial updating task (MUSpace) also correlates quite well with difficult MMM. This suggests that many or most of the participants used a visual or spatial representation that formulates calculations of MMM. This result is consistent with findings that there is a shift from verbal WM toward visuo-spatial WM prediction for multiplication with age. Meyer et al. (2010) found that the central executive and phonological loop components in WM facilitated arithmetic performance during the early stages; however, visuo-spatial WM was more important during later stages. Recently, Soltanlou et al. (2015) also found that multiplication performance correlated with verbal WM in grade 3 but with visuo-spatial WM in grade 4. However, in their study, they focus on simple multiplication problems with multiplicative fact retrieval. In our studies, we focused on multidigit multiplication, which involves other mechanisms than mere multiplication fact retrieval. Participants needed to combine retrieval and procedural operation continuously and efficiently. Under the circumstances, the ability accurately to maintain and update information plays a more important role in MMM tasks. However, the role of the visual or spatial representation in MMM will need to be tested by further experimentation. Notice that the spatial short-term memory task in Experiment 1, Mspace, did not require updating, but simply remembering the location of a series of dots, while MUSpace required changing, that is, updating, a remembered location. The Mspace task did not correlate with MMM.

Previous findings suggest that updating is strongly correlated to mathematical performance (e.g., Andersson, 2008; De Smedt et al., 2009b; Van der Ven et al., 2012; Friso-van den Bos et al., 2013; Cragg and Gilmore, 2014). However, whether domain-general memory updating ability or the capacity for updating specific information is better able to predict mathematical performance is still a controversial issue. In our study, updating appeared to be a common factor relating complex span tasks together: that is, those that were not involved in this process tended not to correlate with MMM performance, whether it involved numbers or not. However, findings from the present study suggest that the relationship between working memory and arithmetic is more complex than previously postulated. Firstly, both the performance of MUcalc and MUSpace correlated with difficult multiplication problems only, but not with easy problems. This indicates that, on one hand, updating memory in these two tasks is particularly relevant for more difficult calculations involving maintenance and updating intermediate numerical results. On the other hand, implies a different memory updating process in MUcalc and MUSpace from MUNumber and MUWord. All the MU tasks required participants to: (1) remember the original information, (2) receive and process the new information, and then (3) replace the original information by the new one. However, the second step in MUcalc and MUSpace was slightly different from MUNumber and MUWord. In the first two tasks, the original information (digit for MUcalc and location for MUSpace) was transferred to the new one by calculation (addition or subtraction for MUcalc, and clockwise turn or counter-clockwise turn for 90° for MUSpace). In contrast, the process in both MUNumber and MUWord was simple comparison (remember larger or smaller one for numerical size or animal size) but not calculation.

Secondly, number updating with calculation (MUcalc) is highly correlated with MMM performance, but word updating (MUWord) is not. This, in turn, suggests a degree of domain-specificity in the updating process, though not in the way originally envisaged by Butterworth (1996) and Iuculano et al. (2011). These studies assumed that the mere presence of numbers in the memory task was critical, while here we found that carrying out arithmetic operations on numbers was what mattered. One may argue that since both the performance of MUcalc and MUSpace were correlated with MMM, the memory updating involved in these two tasks is a domain-general ability. However, although we did find a similar pattern for the correlations between these two updating tasks and the MMM performance, the calculation behind these two tasks could be still different from each other. For example, in the MUSpace task, participants were possibly using a visuo-spatial strategy, a kind of “mental blackboard,” maintaining and manipulating critical information while performing MMM. Evidence shows that the performances of abacus users who have high accuracy and fast speed in mental calculation depend on improved spatial updating ability (Tanaka et al., 2012). However, this requires further investigation.

In summary, our study clearly shows that the processes involved in updating the contents of working memory are critically important to multidigit mental multiplication in adults. However, in order to fully understand the relationship between memory updating and mental arithmetic, we should further investigate the interaction between different memory updating and mathematical tasks for different ages.

### Conflict of interest statement

The authors declare that the research was conducted in the absence of any commercial or financial relationships that could be construed as a potential conflict of interest. The reviewer, Hans-Christoph Nuerk, and handling Editor declared their shared affiliation, and the handling Editor states that the process nevertheless met the standards of a fair and objective review.

## Footnotes

^1^We note that in Experiment 1, the distribution of MUcalc was normal, whereas it turned out to be non-normal (negatively skewed) in Experiment 2, even after arcsine-transformation. This was also the case for MUNumber and MUWord. After arcsine transformation, the distributions were only approximately normal. We speculated that a similar task design (for MUNumber and MUWord, or for MUcalc and MUSpace) in Experiment 2 may yield a learning effect, which led participants to become familiar with those tasks quickly and to easily get better scores in some tasks. For instance, the accuracy rate for MUcalc in Experiment 1 was about 84.2%, and in Experiment 2 it increased to almost 92.6%. Another reason for observing negative skewed distributions in tasks may be that participants who participated in multiplication experiment may regard them as competent for such tasks. If they mistrusted their math ability or hated math at the beginning, they would not have participated in this study. Furthermore, the MUcalc or other MU tasks may be too easy for college students.

^2^We carried out a 2 key press style (a between variable with two levels: experiment 1 and 2) × 2 problem types (a within variable with two levels: 2x1and 4x1) mixed ANOVA on original accuracy and RT respectively. In accuracy, the main effect was only significant for problem types [*F*_(1, 98)_ = 170.984, *p* < 0.001, $\eta_{\text{2}}^{2\mathrm{pt}}$*p* = 0.636]. Participants performed better in type 2 × 1 (*M* = 0.915, *SD* = 0.070) than in type 4 × 1 (*M* = 0.748, *SD* = 0.141) problems. Neither the key press style, nor the interaction between key press style and problem type reached significant, all *p* s = 0.118, which indicated that the way of different key press procedures did not influence participants' accuracy performance. In RT, there was a significant main effect for problem types [*F*_(1, 98)_ = 274.623, *p* < 0.001, $\eta_{\text{p}}^{2}=$ 0.737], which indicated that answering type 2 × 1 (*M* = 6.205, *SD* = 0.201) problem was faster than answering type 4x1 (*M* = 17.989, *SD* = 0.837) problems. Furthermore, both the key press style and their interaction reached significant [*F*_(1, 98)_ = 49.331 and 78.061, both *p* s = 0.001, $\eta_{\text{p}}^{2}=$ 0.335 and 0.443 respectively], which suggested the way of different key press procedures influenced participants' RT performance. The significant main effect for key press style showed that the way of entering exact numbers in Experiment 1 (*M* = 15.568 s, *SD* = 12.076) took longer than the way of using only four buttons to increase or decrease answers in Experiment 2 (*M* = 8.626 s, *SD* = 4.522). In the significant interaction, although there was no RT difference between the way of entering answers for type 2x1 problem in Experiment 1 and 2, the significant difference was revealed between entering type 4x1 problem in Experiment 1 and 2, *p* < 0.001. Answering 4x1 problem by entering exact numbers in Experiment 1 was significantly longer than by pressing only four buttons to reach answers in Experiment 2. Notice that, although RT was faster by pressing only four buttons to reach answer, the RT in Experiment 2 was not correlated with any working memory tasks like those in Experiment 1. It is possible that the participants started pressing the response key without a clear answer in their minds in the Experiment 2, especially when the problems were difficult.

## References

[B1] Andersson U. (2008). Working memory as a predictor of written arithmetical skills in children: the importance of central executive functions. Br. J. Educ. Psychol. 78, 181–203. 10.1348/000709907X209854 17535520

[B2] Andersson U. Lyxell B. (2007). Working memory deficit in children with mathematical difficulties: a general or specific deficit? J. Exp. Child. Psychol. 96, 197–228. 10.1016/j.jecp.2006.10.001 17118398

[B3] Attout L. Fias W. Salmon E. Majerus S. (2014). Common neural substrates for ordinal representation in short-term memory, numerical and alphabetical cognition. PLoS ONE 9:e92049. 10.1371/journal.pone.0092049 24632823 PMC3954845

[B4] Baddeley A. (2003). Working memory: looking back and looking forward. Nat. Rev. Neurosci. 4, 829–839. 10.1038/nrn1201 14523382

[B5] Baddeley A. D. Hitch G. (1977). Commentary on ‘working memory.’ in Human Memory: Basic Processes, ed Bower G. (New York, NY: Academic Press), 191–197.

[B6] Butterworth B. (1996). Short term memory impairment and arithmetical ability. Q. J. Exp. Psychol. A 49, 251–262. 10.1080/713755603 8920104

[B7] Butterworth B. (2003). Dyscalculia Screener. London: nferNelson Pub.

[B8] Butterworth B. (2010). Foundational numerical capacities and the origins of dyscalculia. Trends. Cogn. Sci. 14, 534–541. 10.1016/j.tics.2010.09.007 20971676

[B9] Case R. Kurland D. M. Goldberg J. (1982). Operational efficiency and the growth of short-term memory span. J. Exp. Child. Psychol. 33, 386–404. 10.1016/0022-0965(82)90054-6

[B10] Chandler P. Sweller J. (1991). Cognitive load theory and the format of instruction. Cogn. Instruct. 8, 293–332. 10.1207/s1532690xci0804_2

[B11] Chochon F. Cohen L. Van De Moortele P. Dehaene S. (1999). Differential contributions of the left and right inferior parietal lobules to number processing. J. Cogn. Neurosci. 11, 617–630. 10.1162/089892999563689 10601743

[B12] Cragg L. Gilmore C. (2014). Skills underlying mathematics: the role of executive function in the development of mathematics proficiency. Trends Neurosci. Educ. 3, 63–68. 10.1016/j.tine.2013.12.001

[B13] Daneman M. Carpenter P. A. (1980). Individual differences in working memory and reading. J. Verb. Learn. Verb. Beh. 19, 450–466. 10.1016/S0022-5371(80)90312-6

[B14] De Rammelaere S. Stuyven E. Vandierendonck A. (2001). Verifying simple arithmetic sums and products: are the phonological loop and the central executive involved? Mem. Cogn. 29, 267–273. 10.3758/bf03194920 11352209

[B15] De Smedt B. Janssen R. Bouwens K. Verschaffel L. Boets B. Ghesquière P. (2009a). Working memory and individual differences in mathematics achievement: a longitudinal study from first grade to second grade. J. Exp. Child. Psychol. 103, 186–201. 10.1016/j.jecp.2009.01.004 19281998

[B16] De Smedt B. Verschaffel L. Ghesquière P. (2009b). The predictive value of numerical magnitude comparison for individual differences in mathematics achievement. J. Exp. Child. Psychol. 103, 469–479. 10.1016/j.jecp.2009.01.010 19285682

[B17] Ericsson K. A. Charness N. (1994). Expert performance: its structure and acquisition. Am. Psychol. 49, 725 10.1037/0003-066X.49.8.725

[B18] Ericsson K. A. Kintsch W. (1995). Long-term working memory. Psychol. Rev. 102:211. 10.1037/0033-295X.102.2.211 7740089

[B19] Friso-van den Bos I. van der Ven S. H. Kroesbergen E. H. van Luit J. E. (2013). Working memory and mathematics in primary school children: a meta-analysis. Educ. Res. Rev. 10, 29–44. 10.1016/j.edurev.2013.05.003

[B20] Gathercole S. E. Pickering S. J. Knight C. Stegmann Z. (2004). Working memory skills and educational attainment: evidence from national curriculum assessments at 7 and 14 years of age. Appl. Cogn. Psych. 18, 1–16. 10.1002/acp.934

[B21] Geary D. C. Bailey D. H. Littlefield A. Wood P. Hoard M. K. Nugent L. (2009). First-grade predictors of mathematical learning disability: a latent class trajectory analysis. Cogn. Dev. 24, 411–429. 10.1016/j.cogdev.2009.10.001 20046817 PMC2813681

[B22] Geary D. C. Hoard M. K. Byrd-Craven J. Nugent L. Numtee C. (2007). Cognitive mechanisms underlying achievement deficits in children with mathematical learning disability. Child Dev. 78, 1343–1359. 10.1111/j.1467-8624.2007.01069.x 17650142 PMC4439199

[B23] Grabner R. H. Ansari D. Koschutnig K. Reishofer G. Ebner F. Neuper C. (2009). To retrieve or to calculate? Left angular gyrus mediates the retrieval of arithmetic facts during problem solving. Neuropsychologia 47, 604–608. 10.1016/j.neuropsychologia.2008.10.013 19007800

[B24] Hecht S. A. (2002). Counting on working memory in simple arithmetic when counting is used for problem solving. Mem. Cogn. 30, 447–455. 10.3758/BF03194945 12061765

[B25] Hunter I. M. (1962). An exceptional talent for calculative thinking. Br. J. Psychol. 53, 243–258. 10.1111/j.2044-8295.1962.tb00831.x 14450055

[B26] Iuculano T. Moro R. Butterworth B. (2011). Updating Working Memory and arithmetical attainment in school. Learn. Individ. Differ. 21, 655–661. 10.1016/j.lindif.2010.12.002

[B27] Kovas Y. Haworth C. Dale P. Plomin R. (2007). The genetic and environmental origins of learning abilities and disabilities in the early school years. Monogr. Soc. Res. Child. Dev. 72, 1–156. Available online at: http://www.jstor.org/stable/30163178 17995572 10.1111/j.1540-5834.2007.00439.x PMC2784897

[B28] Lee K. M. (2000). Cortical areas differentially involved in multiplication and subtraction: a functional magnetic resonance imaging study and correlation with a case of selective acalculia. Ann. Neurol. 48, 657–661. 10.1002/1531-8249(200010)48:4<657::AID-ANA13>3.0.CO;2-K 11026450

[B29] Lewandowsky S. Oberauer K. Yang L.-X. Ecker U. K. (2010). A working memory test battery for MATLAB. Behav. Res. Meth. 42, 571–585. 10.3758/BRM.42.2.571 20479189

[B30] Logie R. H. Gilhooly K. J. Wynn V. (1994). Counting on working memory in arithmetic problem solving. Mem. Cogn. 22, 395–410. 10.3758/BF03200866 7934946

[B31] Lucchelli F. De Renzi E. (1993). Primary dyscalculia after a medial frontal lesion of the left hemisphere. J. Neurol. Neurosur. Psychiatry 56, 304–307. 10.1136/jnnp.56.3.304 7681473 PMC1014868

[B32] McLean J. F. Hitch G. J. (1999). Working memory impairments in children with specific arithmetical difficulties. J. Exp. Child. Psychol. 74, 240–260. 10.1006/jecp.1999.2516 10527556

[B33] Meyer M. Salimpoor V. Wu S. Geary D. Menon V. (2010). Differential contribution of specific working memory components to mathematics achievement in 2nd and 3rd graders. Learn. Individ. Differ. 20, 101–109. 10.1016/j.lindif.2009.08.004 21660238 PMC3109434

[B34] Pickering S. Gathercole S. E. (2001). Working Memory test Battery for Children (WMTB-C). London: Psychological Corporation Europe.

[B35] Siegel L. S. Ryan E. B. (1989). The development of working memory in normally achieving and subtypes of learning disabled children. Child Dev. 60, 973–980. 10.2307/1131037 2758890

[B36] Soltanlou M. Pixner S. Nuerk H.-C. (2015). Contribution of working memory in multiplication fact network in children may shift from verbal to visuo-spatial: a longitudinal investigation. Front. Psychol. 6:e1062. 10.3389/fpsyg.2015.01062 26257701 PMC4512035

[B37] Tanaka S. Seki K. Hanakawa T. Harada M. Sugawara S. K. Sadato N.. (2012). Abacus in the brain: a longitudinal functional MRI study of a skilled abacus user with a right hemispheric lesion. Front. Psychol. 3:315. 10.3389/fpsyg.2012.00315 22969743 PMC3428809

[B38] Tuovinen J. E. Sweller J. (1999). A comparison of cognitive load associated with discovery learning and worked examples. J. Educ. Psychol. 91:334 10.1037/0022-0663.91.2.334

[B39] Van der Ven S. H. Kroesbergen E. H. Boom J. Leseman P. P. (2012). The development of executive functions and early mathematics: a dynamic relationship. Br. J. Educ. Psychol. 82, 100–119. 10.1111/j.2044-8279.2011.02035.x 22429060

[B40] Zamarian L. Ischebeck A. Delazer M. (2009). Neuroscience of learning arithmetic—evidence from brain imaging studies. Neurosci. Biobehav. Rev. 33, 909–925. 10.1016/j.neubiorev.2009.03.005 19428500

