# Supplementary material for: Memory Updating and Mental Arithmetic
Source: Front Psychol. 2016 Feb 2;7:72. doi: 10.3389/fpsyg.2016.00072 (PMC4735758; doi:10.3389/fpsyg.2016.00072)
Supplement: Supplementary file 1 [file DataSheet1.PDF]

Supplementary data distribution

In Experiment 1, to investigate the correlation between working memory and multidigit mental multiplication (MMM), we arcsine-transformed the data distribution of 3 working memory tasks (MUcalc, Mletter, and Mspace) for carrying out the correlation and multiple regressions analyses. The p value of normality testing of each task after arcsine-transformation is respectively depicted (according to Shapiro-Wilk). Arcsine-MUcalc, $p = .234$; Arcsine-Mletter, $p = .427$; Arcsine-Mspace, $p = .299$. The original data distribution for accuracy and RT of MMM was normal, $p = .237$ and .528 respectively.

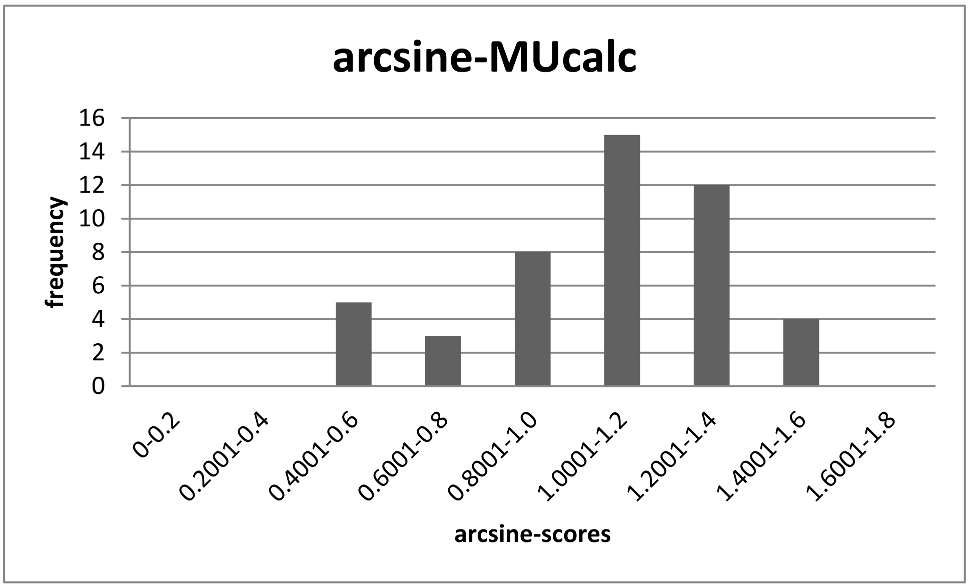

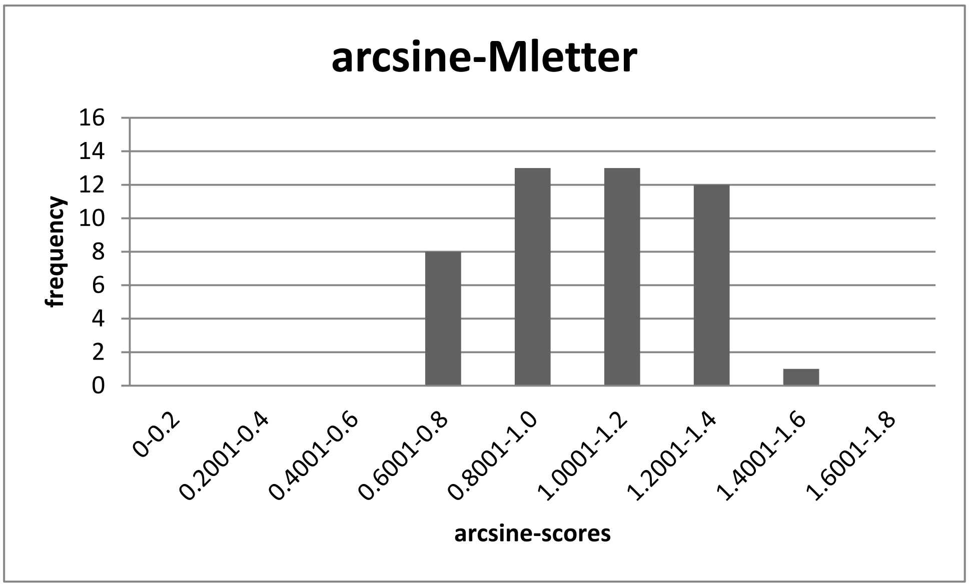

arcsine-Mletter
16
14
12
10
8
6
4
2
0
frequency
0-0.2
0.2001-0.4
0.4001-0.6
0.6001-0.8
0.8001-1.0
1.0001-1.2
1.2001-1.4
1.4001-1.6
1.6001-1.8
arcsine-scores

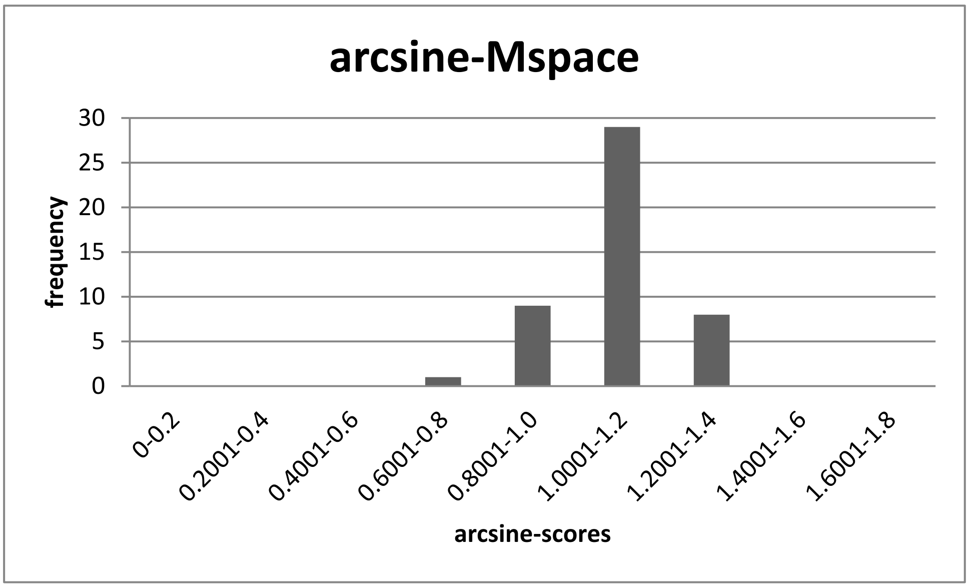

arcsine-Mspace
30
25
20
15
10
5
0
frequency
0-0.2
0.2001-0.4
0.4001-0.6
0.6001-0.8
0.8001-1.0
1.0001-1.2
1.2001-1.4
1.4001-1.6
1.6001-1.8
arcsine-scores

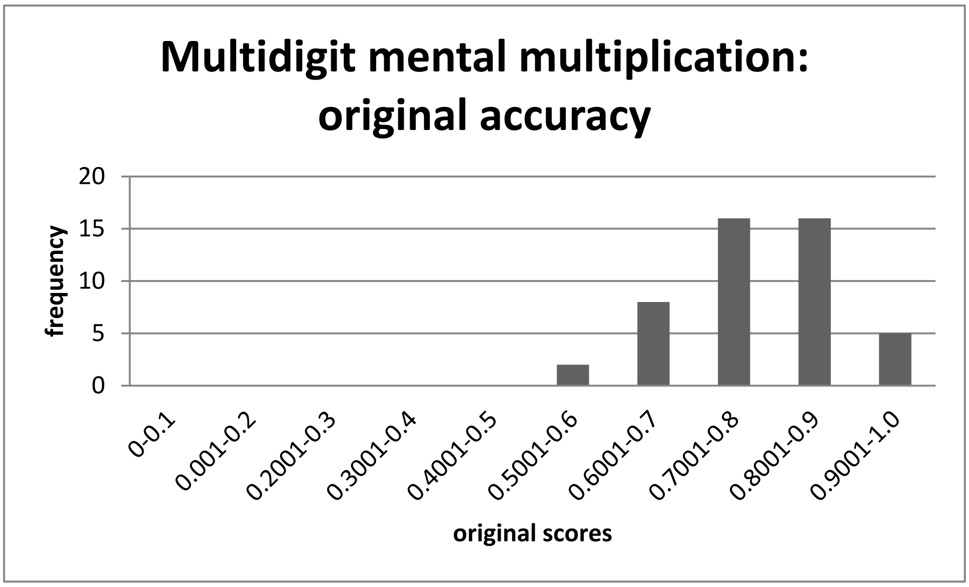
Multidigit mental multiplication: original accuracy
20
15
10
5
0
frequency
0-0.1
0.001-0.2
0.2001-0.3
0.3001-0.4
0.4001-0.5
0.5001-0.6
0.6001-0.7
0.7001-0.8
0.8001-0.9
0.9001-1.0
original scores

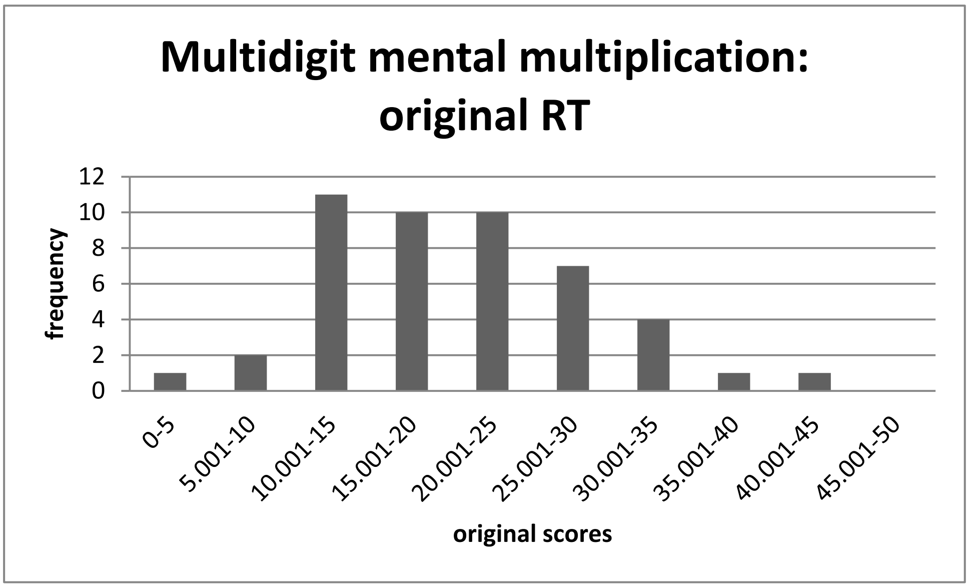
Multidigit mental multiplication: original RT
12
10
8
6
4
2
0
frequency
0-5
5.001-10
10.001-15
15.001-20
20.001-25
25.001-30
30.001-35
35.001-40
40.001-45
45.001-50
original scores

In Experiment 2, for investigating the correlation between memory updating and multidigit mental multiplication (MMM), we arcsine-transformed the data distribution of 4 memory updating tasks (MUcalc, MUSpace, MUNumber, and MUWord) and the accuracies of MMM for carrying out the correlation and multiple regressions analyses. The p value of normality testing of each task after arcsine-transformation is respectively depicted (according to Kolmogorov-Smirnov). Arcsine-MUcalc, $p < .001$; Arcsine-MUSpace: $p = .059$; Arcsine-MUNumber: $p = .035$; Arcsine- MUWord: $p = .048$. Arcsine-Multiplication (all): $p = .200$; Arcsine-Multiplication (2x1): $p = .059$; Arcsine-Multiplication (4x1): $p = .063$. The original data distributions of RT of MMM (all, 2x1, and 4x1) were all normal, all ps = .200.

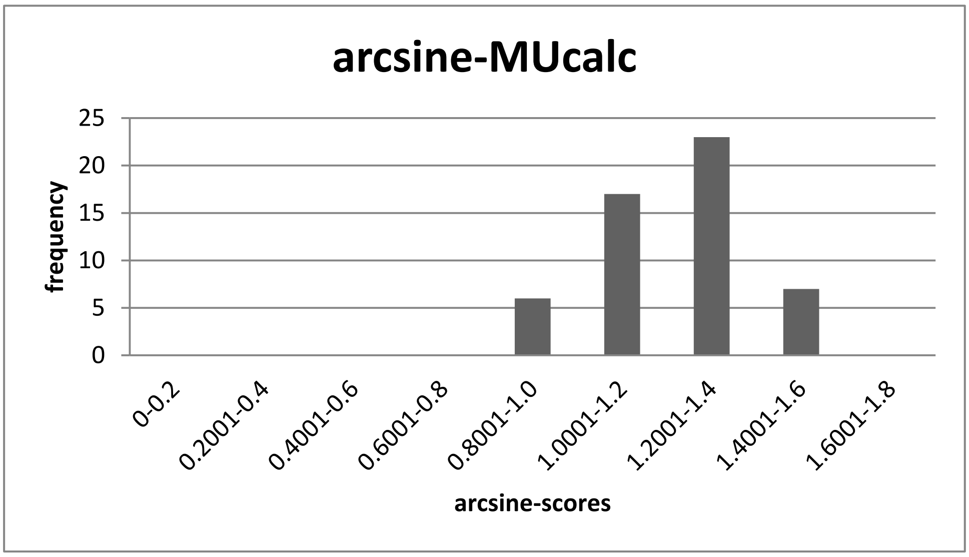

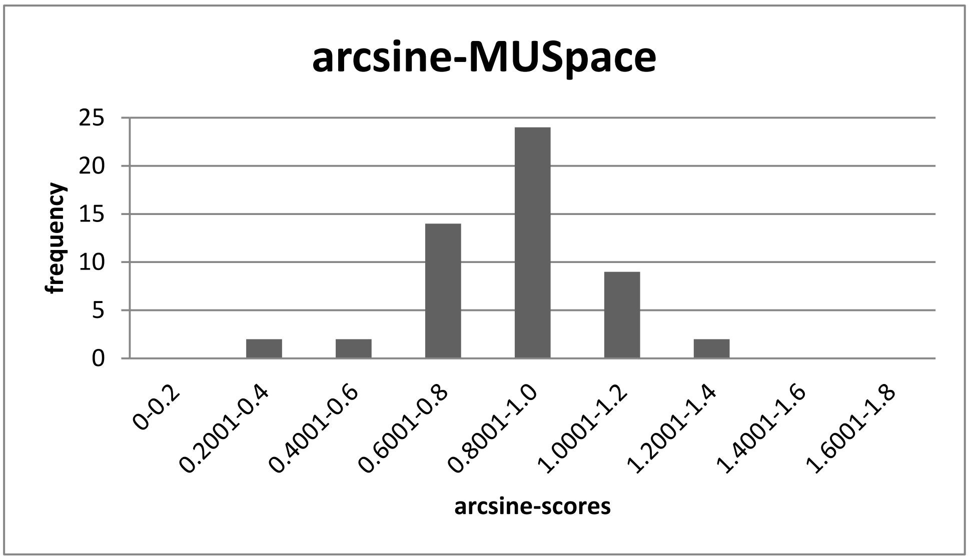
arcsine-MUSpace
frequency
25
20
15
10
5
0
0-0.2
0.2001-0.4
0.4001-0.6
0.6001-0.8
0.8001-1.0
1.0001-1.2
1.2001-1.4
1.4001-1.6
1.6001-1.8
arcsine-scores

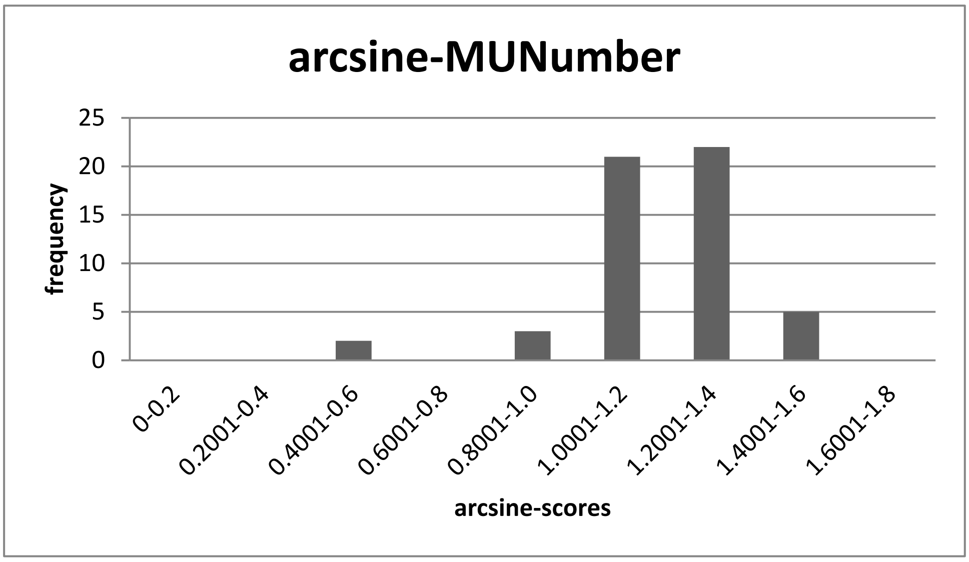
arcsine-MUNumber
frequency
25
20
15
10
5
0
0-0.2
0.2001-0.4
0.4001-0.6
0.6001-0.8
0.8001-1.0
1.0001-1.2
1.2001-1.4
1.4001-1.6
1.6001-1.8
arcsine-scores

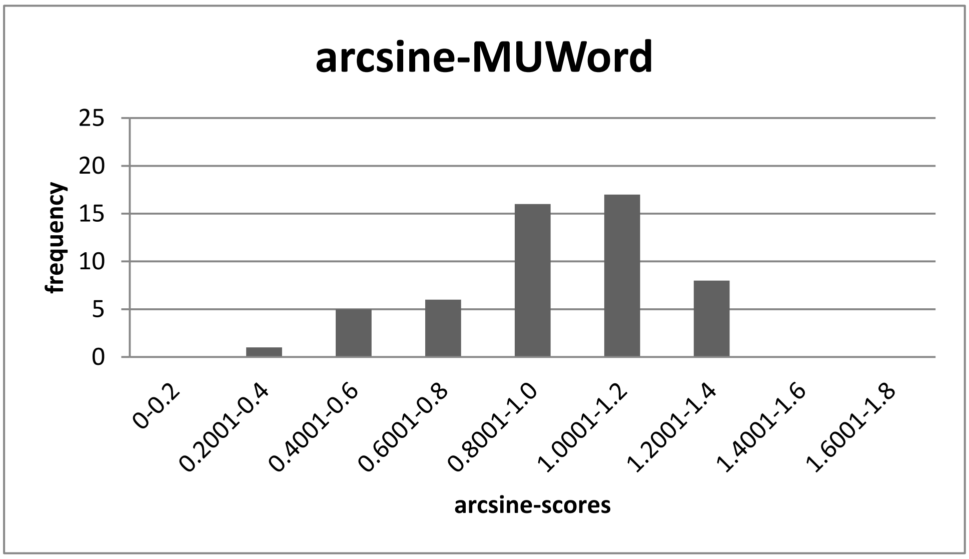
arcsine-MUWord
frequency
25
20
15
10
5
0
0-0.2
0.2001-0.4
0.4001-0.6
0.6001-0.8
0.8001-1.0
1.0001-1.2
1.2001-1.4
1.4001-1.6
1.6001-1.8
arcsine-scores

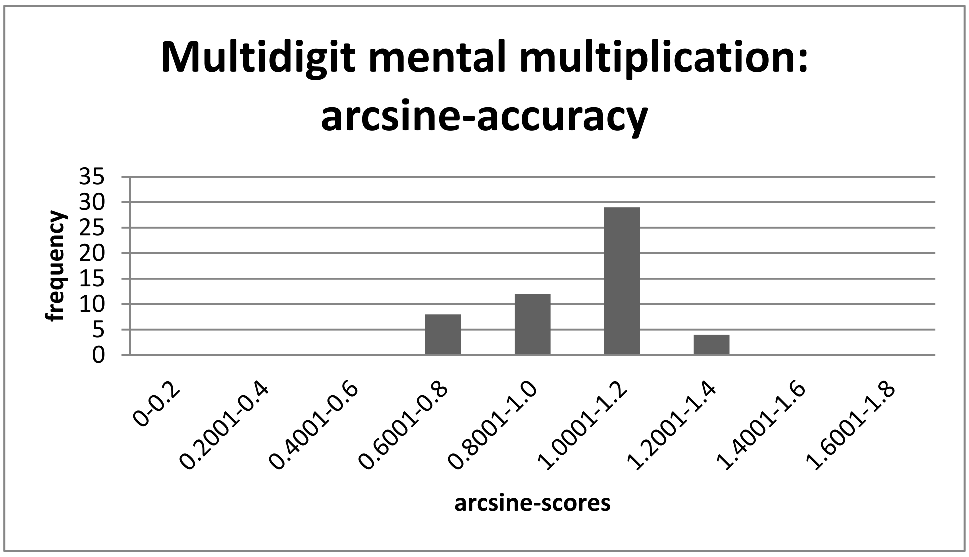

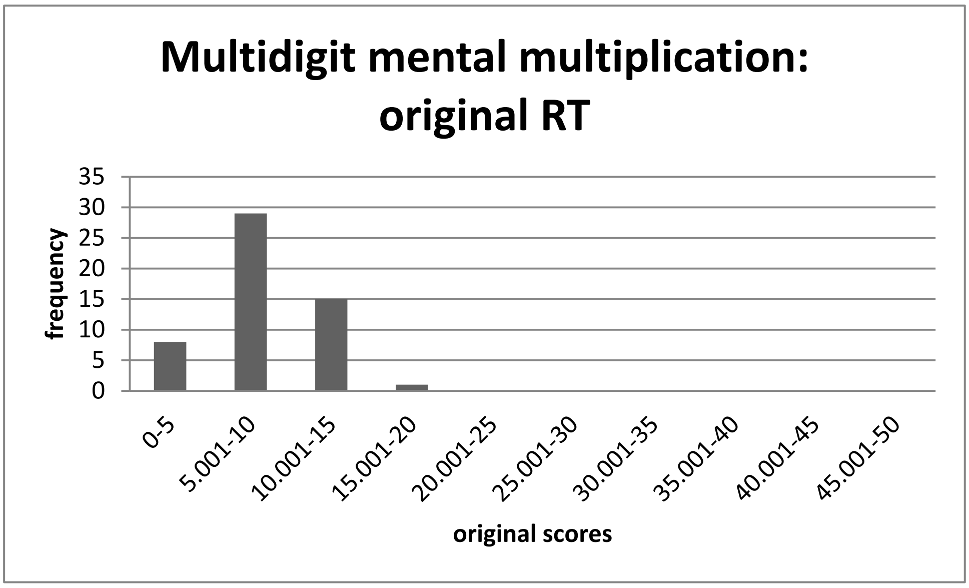

Note. We note that in Experiment 1, the distribution of MUcalc was normal, whereas it turned out to be non-normal (negatively skewed) in Experiment 2, even after arcsine-transformation. This was also the case for MUNumber and MUWord. After arcsine transformation, the distributions were only approximately normal. We speculated that a similar task design (for MUNumber and MUWord, or for MUcalc and MUSpace) in Experiment 2 may yield a learning effect, which led participants to

become familiar with those tasks quickly and to easily get better scores in some tasks. For instance, the accuracy rate for MUcalc in Experiment 1 was about 84.2%, and in Experiment 2 it increased to almost 92.6%. Another reason for observing negative skewed distributions in tasks may be that participants who participated in multiplication experiment may regard them as competent for such tasks. If they mistrusted their math ability or hated math at the beginning, they would not have participated in this study. Furthermore, the MUcalc or other MU tasks may be too easy for college students.
