# Supplementary material for: Memory Updating and Mental Arithmetic
Source: Front Psychol. 2016 Feb 2;7:72. doi: 10.3389/fpsyg.2016.00072 (PMC4735758; doi:10.3389/fpsyg.2016.00072)
Supplement: Supplementary file 2 [file DataSheet2.docx]

**Appendix 1**

**In Experiment 1: All of the four types of multidigit multiplication problems and their correct answers.**

*Note.* In Experiment 1, participants were assigned 80 problems in total, including type 2x1, 3x1, 4x1, and 2x2 conditions, in which were 20 problems respectively. The problems of each condition were randomly selected from this item pool by the matlab (2008b) program. The total 80 problems that every participant solved in the experiment were not necessarily the same.

| **Type 2x1 problem** | | | | | |
| --- | --- | --- | --- | --- | --- |
| 23x4=92 | 32x7=224 | 42x6=252 | 52x6=312 | 62x7=434 | 72x8=576 |
| 23x5=115 | 32x8=256 | 42x7=294 | 52x7=364 | 62x8=496 | 72x9=648 |
| 23x6=138 | 32x9=288 | 42x8=336 | 52x8=416 | 62x9=558 | 73x4=292 |
| 23x7=161 | 34x5=170 | 42x9=378 | 52x9=468 | 63x4=252 | 73x5=365 |
| 23x8=184 | 34x6=204 | 43x5=215 | 53x4=212 | 63x5=315 | 73x6=438 |
| 23x9=207 | 34x7=238 | 43x6=258 | 53x6=318 | 63x7=441 | 73x8=584 |
| 24x3=72 | 34x8=272 | 43x7=301 | 53x7=371 | 63x8=504 | 73x9=657 |
| 24x5=120 | 34x9=306 | 43x8=344 | 53x8=424 | 63x9=567 | 74x3=222 |
| 24x6=144 | 35x2=70 | 43x9=387 | 53x9=477 | 64x3=192 | 74x5=370 |
| 24x7=168 | 35x4=140 | 45x2=90 | 54x3=162 | 64x5=320 | 74x6=444 |
| 24x8=192 | 35x6=210 | 45x3=135 | 54x6=324 | 64x7=448 | 74x8=592 |
| 24x9=216 | 35x7=245 | 45x6=270 | 54x7=378 | 64x8=512 | 74x9=666 |
| 25x3=75 | 35x8=280 | 45x7=315 | 54x8=432 | 64x9=576 | 75x2=150 |
| 25x6=150 | 35x9=315 | 45x8=360 | 54x9=486 | 65x2=130 | 75x3=225 |
| 25x7=175 | 36x2=72 | 45x9=405 | 56x2=112 | 65x3=195 | 75x4=300 |
| 25x8=200 | 36x4=144 | 46x2=92 | 56x3=168 | 65x4=260 | 75x6=450 |
| 25x9=225 | 36x5=180 | 46x3=138 | 56x4=224 | 65x7=455 | 75x8=600 |
| 26x3=78 | 36x7=252 | 46x5=230 | 56x7=392 | 65x8=520 | 75x9=675 |
| 26x5=130 | 36x8=288 | 46x7=322 | 56x8=448 | 65x9=585 | 76x2=152 |
| 26x7=182 | 36x9=324 | 46x8=368 | 56x9=504 | 67x2=134 | 76x3=228 |
| 26x8=208 | 37x2=74 | 46x9=414 | 57x2=114 | 67x3=201 | 76x4=304 |
| 26x9=234 | 37x4=148 | 47x2=94 | 57x3=171 | 67x4=268 | 76x5=380 |
| 27x3=81 | 37x5=185 | 47x3=141 | 57x4=228 | 67x5=335 | 76x5=380 |
| 27x5=135 | 37x6=222 | 47x5=235 | 57x6=342 | 67x8=536 | 76x8=608 |
| 27x6=162 | 37x8=296 | 47x6=282 | 57x8=456 | 67x9=603 | 76x9=684 |
| 27x8=216 | 37x9=333 | 47x8=376 | 57x9=513 | 68x2=136 | 78x2=156 |
| 27x9=243 | 38x2=76 | 47x9=423 | 58x2=116 | 68x3=204 | 78x3=234 |
| 28x3=84 | 38x4=152 | 48x2=96 | 58x3=174 | 68x4=272 | 78x4=312 |
| 28x5=140 | 38x5=190 | 48x3=144 | 58x4=232 | 68x5=340 | 78x5=390 |
| 28x6=168 | 38x6=228 | 48x5=240 | 58x6=348 | 68x7=476 | 78x6=468 |
| 28x7=196 | 38x7=266 | 48x6=288 | 58x7=406 | 68x9=612 | 78x9=702 |
| 28x9=252 | 38x9=342 | 48x7=336 | 58x9=522 | 69x2=138 | 79x2=158 |
| 29x3=87 | 39x2=78 | 48x9=432 | 59x2=118 | 69x3=207 | 79x3=237 |
| 29x5=145 | 39x4=156 | 49x2=98 | 59x3=177 | 69x4=276 | 79x4=316 |
| 29x6=174 | 39x5=195 | 49x3=147 | 59x4=236 | 69x5=345 | 79x5=395 |
| 29x7=203 | 39x6=234 | 49x5=245 | 59x6=354 | 69x7=483 | 79x6=474 |
| 29x8=232 | 39x7=273 | 49x6=294 | 59x7=413 | 69x8=552 | 79x8=632 |
| 32x5=160 | 39x8=312 | 49x7=343 | 59x8=472 | 72x5=360 | 82x5=410 |
| 32x6=192 | 42x5=210 | 49x8=392 | 62x5=310 | 72x6=432 | 82x6=492 |
| 82x7=574 | 85x3=255 | 87x4=348 | 92x8=736 | 95x4=380 | 97x5=485 |
| 82x9=738 | 85x4=340 | 87x5=435 | 93x4=372 | 95x6=570 | 97x6=582 |
| 83x4=332 | 85x6=510 | 87x6=522 | 93x5=465 | 95x7=665 | 97x8=776 |
| 83x5=415 | 85x7=595 | 87x9=783 | 93x6=558 | 95x8=760 | 98x2=196 |
| 83x6=498 | 85x9=765 | 89x2=178 | 93x7=651 | 96x2=192 | 98x3=294 |
| 83x7=581 | 86x2=172 | 89x3=267 | 93x8=744 | 96x3=288 | 98x4=392 |
| 83x9=747 | 86x3=258 | 89x4=356 | 94x3=282 | 96x4=384 | 98x5=490 |
| 84x3=252 | 86x4=344 | 89x5=445 | 94x5=470 | 96x5=480 | 98x6=588 |
| 84x5=420 | 86x5=430 | 89x6=534 | 94x6=564 | 96x7=672 | 98x7=686 |
| 84x6=504 | 86x7=602 | 89x7=623 | 94x7=658 | 96x8=768 |  |
| 84x7=588 | 86x9=774 | 92x5=460 | 94x8=752 | 97x2=194 |  |
| 84x9=756 | 87x2=174 | 92x6=552 | 95x2=190 | 97x3=291 |  |
| 85x2=170 | 87x3=261 | 92x7=644 | 95x3=285 | 97x4=388 |  |

| **Type 3x1 problem** | | | | | |
| --- | --- | --- | --- | --- | --- |
| 234x5=1170 | 284x7=1988 | 346x8=2768 | 385x9=3465 | 437x2=874 | 476x3=1428 |
| 235x7=1645 | 285x7=1995 | 347x2=694 | 387x9=3483 | 437x8=3496 | 476x5=2380 |
| 237x5=1185 | 285x9=2565 | 347x6=2082 | 389x4=1556 | 438x2=876 | 476x9=4284 |
| 237x8=1896 | 286x9=2574 | 348x2=696 | 392x5=1960 | 438x7=3066 | 479x3=1437 |
| 238x7=1666 | 293x6=1758 | 349x2=698 | 392x7=2744 | 439x2=878 | 479x5=2395 |
| 243x9=2187 | 294x5=1470 | 349x6=2094 | 394x5=1970 | 439x7=3073 | 482x7=3374 |
| 246x8=1968 | 294x6=1764 | 349x8=2792 | 394x6=2364 | 452x9=4068 | 482x9=4338 |
| 247x6=1482 | 294x7=2058 | 354x7=2478 | 394x7=2758 | 453x7=3171 | 483x6=2898 |
| 249x6=1494 | 294x8=2352 | 356x7=2492 | 395x4=1580 | 456x3=1368 | 483x7=3381 |
| 249x8=1992 | 295x7=2065 | 362x9=3258 | 395x6=2370 | 456x7=3192 | 483x9=4347 |
| 254x9=2286 | 297x5=1485 | 364x9=3276 | 395x7=2765 | 457x3=1371 | 485x3=1455 |
| 256x7=1792 | 297x6=1782 | 365x4=1460 | 395x8=3160 | 458x3=1374 | 486x3=1458 |
| 263x9=2367 | 298x5=1490 | 367x4=1468 | 398x4=1592 | 459x3=1377 | 486x9=4374 |
| 264x7=1848 | 324x7=2268 | 368x7=2576 | 398x7=2786 | 462x7=3234 | 487x9=4383 |
| 265x7=1855 | 324x8=2592 | 372x5=1860 | 423x6=2538 | 462x8=3696 | 492x5=2460 |
| 265x9=2385 | 326x7=2282 | 372x6=2232 | 425x7=2975 | 463x7=3241 | 492x6=2952 |
| 273x8=2184 | 327x6=1962 | 374x6=2244 | 427x6=2562 | 465x9=4185 | 493x6=2958 |
| 273x9=2457 | 328x6=1968 | 374x9=3366 | 428x7=2996 | 468x7=3276 | 496x7=3472 |
| 274x5=1370 | 328x7=2296 | 375x6=2250 | 429x6=2574 | 469x7=3283 | 497x6=2982 |
| 274x9=2466 | 329x6=1974 | 375x9=3375 | 432x5=2160 | 472x9=4248 | 497x8=3976 |
| 275x6=1650 | 342x7=2394 | 378x5=1890 | 432x6=2592 | 473x5=2365 | 498x5=2490 |
| 276x5=1380 | 342x8=2736 | 379x6=2274 | 432x7=3024 | 473x6=2838 | 498x7=3486 |
| 278x6=1668 | 345x2=690 | 382x6=2292 | 435x2=870 | 473x8=3784 | 523x6=3138 |
| 279x5=1395 | 345x6=2070 | 382x7=2674 | 435x7=3045 | 473x9=4257 | 523x7=3661 |
| 283x7=1981 | 345x8=2760 | 384x9=3456 | 435x8=3480 | 475x3=1425 | 524x6=3144 |
| 283x9=2547 | 346x2=692 | 385x4=1540 | 436x2=872 | 475x6=2850 | 526x7=3682 |
| 527x6=3162 | 586x4=2344 | 675x3=2025 | 743x6=4458 | 823x6=4938 | 879x2=1758 |
| 528x6=3168 | 587x2=1174 | 678x2=1356 | 743x9=6687 | 823x7=5761 | 879x3=2637 |
| 532x6=3192 | 587x3=1761 | 678x3=2034 | 745x3=2235 | 824x6=4944 | 879x5=4395 |
| 532x7=3724 | 589x2=1178 | 678x5=3390 | 745x6=4470 | 824x7=5768 | 892x5=4460 |
| 532x8=4256 | 589x4=2356 | 683x4=2732 | 746x3=2238 | 825x7=5775 | 892x6=5352 |
| 532x9=4788 | 593x6=3558 | 683x9=6147 | 746x8=5968 | 826x7=5782 | 894x5=4470 |
| 534x7=3738 | 593x7=4151 | 684x9=6156 | 748x6=4488 | 829x6=4974 | 894x6=5364 |
| 534x8=4272 | 594x6=3564 | 685x3=2055 | 749x3=2247 | 832x5=4160 | 894x7=6258 |
| 536x4=2144 | 594x7=4158 | 685x7=4795 | 758x3=2274 | 835x4=3340 | 895x4=3580 |
| 536x8=4288 | 594x8=4752 | 685x9=6165 | 759x3=2277 | 836x7=5852 | 895x6=5370 |
| 537x8=4296 | 596x2=1192 | 687x2=1374 | 762x9=6858 | 837x5=4185 | 896x2=1792 |
| 539x4=2156 | 596x8=4768 | 687x3=2061 | 763x4=3052 | 842x6=5052 | 896x5=4480 |
| 542x7=3794 | 597x2=1194 | 687x4=2748 | 765x4=3060 | 842x7=5894 | 896x7=6272 |
| 542x8=4336 | 597x6=3582 | 692x5=3460 | 765x9=6885 | 843x6=5058 | 897x4=3588 |
| 542x9=4878 | 597x8=4776 | 692x7=4844 | 768x2=1536 | 845x3=2535 | 923x6=5538 |
| 543x6=3258 | 623x7=4361 | 693x4=2772 | 768x4=3072 | 846x3=2538 | 923x7=6461 |
| 543x9=4887 | 624x7=4368 | 693x5=3465 | 782x6=4692 | 849x3=2547 | 924x6=5544 |
| 546x8=4368 | 632x5=3160 | 693x7=4851 | 782x9=7038 | 849x6=5094 | 925x6=5550 |
| 547x3=1641 | 632x8=5056 | 694x3=2082 | 783x4=3132 | 852x7=5964 | 926x7=6482 |
| 547x6=3282 | 632x9=5688 | 694x5=3470 | 783x9=7047 | 854x7=5978 | 927x6=5562 |
| 548x3=1644 | 634x7=4438 | 694x8=5552 | 785x3=2355 | 856x3=2568 | 928x7=6496 |
| 549x8=4392 | 634x8=5072 | 695x4=2780 | 785x4=3140 | 856x7=5992 | 932x5=4660 |
| 562x7=3934 | 635x8=5080 | 695x7=4865 | 786x3=2358 | 857x3=2571 | 932x7=6524 |
| 563x7=3941 | 637x8=5096 | 695x8=5560 | 786x4=3144 | 862x7=6034 | 932x8=7456 |
| 567x4=2268 | 638x4=2552 | 697x2=1394 | 786x9=7074 | 862x9=7758 | 935x4=3740 |
| 569x4=2276 | 638x5=3190 | 697x4=2788 | 789x2=1578 | 863x4=3452 | 936x5=4680 |
| 569x7=3983 | 639x4=2556 | 698x2=1396 | 792x8=6336 | 863x7=6041 | 936x7=6552 |
| 572x6=3432 | 639x7=4473 | 723x6=4338 | 793x5=3965 | 863x9=7767 | 936x8=7488 |
| 572x8=4576 | 643x9=5787 | 723x8=5784 | 793x6=4758 | 864x7=6048 | 937x8=7496 |
| 572x9=5148 | 647x3=1941 | 724x6=4344 | 793x8=6344 | 865x2=1730 | 942x7=6594 |
| 573x8=4584 | 647x8=5176 | 724x8=5792 | 794x3=2382 | 865x3=2595 | 943x6=5658 |
| 573x9=5157 | 649x3=1947 | 728x6=4368 | 794x5=3970 | 865x9=7785 | 943x8=7544 |
| 574x3=1722 | 649x8=5192 | 732x5=3660 | 795x2=1590 | 869x2=1738 | 945x3=2835 |
| 574x6=3444 | 652x9=5868 | 732x8=5856 | 795x3=2385 | 873x6=5238 | 945x6=5670 |
| 574x8=4592 | 653x9=5877 | 734x5=3670 | 795x4=3180 | 874x6=5244 | 945x8=7560 |
| 576x3=1728 | 657x3=1971 | 735x4=2940 | 795x6=4770 | 874x9=7866 | 946x3=2838 |
| 576x9=5184 | 672x8=5376 | 735x8=5880 | 796x3=2388 | 875x3=2625 | 946x8=7568 |
| 579x3=1737 | 672x9=6048 | 738x5=3690 | 796x5=3980 | 875x9=7875 | 947x8=7576 |
| 579x6=3474 | 674x5=3370 | 739x4=2956 | 798x3=2394 | 876x2=1752 | 948x6=5688 |
| 582x6=3492 | 674x8=5392 | 739x5=3695 | 798x4=3192 | 876x3=2628 | 953x7=6671 |
| 586x3=1758 | 675x2=1350 | 742x8=5936 | 798x6=4788 | 876x5=4380 | 954x7=6678 |
| 962x8=7696 | 967x2=1934 | 972x6=5832 | 974x8=7792 | 983x6=5898 | 985x3=2955 |
| 963x7=6741 | 967x4=3868 | 972x8=7776 | 975x2=1950 | 983x7=6881 | 986x3=2958 |
| 965x3=2895 | 968x2=1936 | 973x8=7784 | 976x5=4880 | 984x3=2952 | 986x4=3944 |
| 965x4=3860 | 968x4=3872 | 974x3=2922 | 978x2=1956 | 984x7=6888 |  |
| 965x7=6755 | 972x5=4860 | 974x6=5844 | 978x3=2934 | 985x2=1970 |  |

| **Type 4x1 problem** | | | | | | |
| --- | --- | --- | --- | --- | --- | --- |
| 2345x8=18760 | 2974x8=23792 | 3524x7=24668 | 4327x6=25962 | 4562x7=31934 | 4895x3=14685 |  |
| 2374x5=11870 | 2976x5=14880 | 3568x7=24976 | 4328x7=30296 | 4563x7=31941 | 4896x3=14688 |  |
| 2376x5=11880 | 3247x8=25976 | 3625x7=25375 | 4329x6=25974 | 4579x3=13737 | 4923x7=34461 |  |
| 2384x7=16688 | 3256x7=22792 | 3642x7=25494 | 4356x2=8712 | 4597x3=13791 | 4936x7=34552 |  |
| 2385x7=16695 | 3264x7=22848 | 3654x7=25578 | 4357x2=8714 | 4623x7=32361 | 4938x5=24690 |  |
| 2394x5=11970 | 3278x6=19668 | 3685x4=14740 | 4358x2=8716 | 4632x9=41688 | 4957x3=14871 |  |
| 2394x7=16758 | 3279x6=19674 | 3687x4=14748 | 4359x2=8718 | 4638x7=32466 | 4958x3=14874 |  |
| 2395x7=16765 | 3298x6=19788 | 3689x4=14756 | 4362x7=30534 | 4657x3=13971 | 4965x3=14895 |  |
| 2396x5=11980 | 3427x6=20562 | 3694x7=25858 | 4365x2=8730 | 4682x7=32774 | 4972x6=29832 |  |
| 2398x5=11990 | 3456x2=6912 | 3697x4=14788 | 4365x7=30555 | 4685x7=32795 | 4973x5=24865 |  |
| 2435x8=19480 | 3457x2=6914 | 3724x6=22344 | 4367x2=8734 | 4698x7=32886 | 4975x3=14925 |  |
| 2473x6=14838 | 3458x2=6916 | 3729x6=22374 | 4368x2=8736 | 4728x6=28368 | 4976x3=14928 |  |
| 2539x7=17773 | 3459x2=6918 | 3742x6=22452 | 4368x7=30576 | 4735x8=37880 | 4982x6=29892 |  |
| 2543x9=22887 | 3465x2=6930 | 3749x8=29992 | 4369x2=8738 | 4762x9=42858 | 4985x7=34895 |  |
| 2568x7=17976 | 3467x2=6934 | 3765x9=33885 | 4372x8=34976 | 4763x9=42867 | 4986x3=14958 |  |
| 2643x9=23787 | 3468x2=6936 | 3792x5=18960 | 4375x2=8750 | 4782x6=28692 | 5246x8=41968 |  |
| 2685x7=18795 | 3469x2=6938 | 3842x7=26894 | 4376x2=8752 | 4783x6=28698 | 5249x6=31494 |  |
| 2739x5=13695 | 3475x2=6950 | 3854x7=26978 | 4378x2=8756 | 4785x3=14355 | 5249x8=41992 |  |
| 2745x8=21960 | 3476x2=6952 | 3924x7=27468 | 4378x5=21890 | 4789x3=14367 | 5268x7=36876 |  |
| 2749x8=21992 | 3478x2=6956 | 3925x6=23550 | 4379x2=8758 | 4792x5=23960 | 5327x6=31962 |  |
| 2754x9=24786 | 3479x2=6958 | 3942x8=31536 | 4382x7=30674 | 4793x5=23965 | 5328x7=37296 |  |
| 2765x9=24885 | 3479x6=20874 | 3945x8=31560 | 4385x2=8770 | 4793x6=28758 | 5329x6=31974 |  |
| 2793x6=16758 | 3482x6=20892 | 3947x6=23682 | 4386x2=8772 | 4795x3=14385 | 5346x8=42768 |  |
| 2794x6=16764 | 3485x2=6970 | 3964x7=27748 | 4387x2=8774 | 4798x5=23990 | 5362x7=37534 |  |
| 2854x7=19978 | 3486x2=6972 | 3974x8=31792 | 4389x2=8778 | 4829x6=28974 | 5368x7=37576 |  |
| 2854x9=25686 | 3487x2=6974 | 3978x5=19890 | 4392x7=30744 | 4832x9=43488 | 5369x7=37583 |  |
| 2864x9=25776 | 3489x2=6978 | 3982x7=27874 | 4395x2=8790 | 4839x7=33873 | 5386x4=21544 |  |
| 2934x5=14670 | 3492x8=27936 | 3987x4=15948 | 4396x2=8792 | 4853x7=33971 | 5394x7=37758 |  |
| 2938x5=14690 | 3495x2=6990 | 4235x7=29645 | 4396x7=30772 | 4853x9=43677 | 5396x4=21584 |  |
| 2947x8=23576 | 3495x8=27960 | 4275x6=25650 | 4397x2=8794 | 4856x3=14568 | 5423x7=37961 |  |
| 2948x6=17688 | 3496x2=6992 | 4278x6=25668 | 4398x2=8796 | 4859x3=14577 | 5428x6=32568 |  |
| 2964x7=20748 | 3497x2=6994 | 4283x6=25698 | 4398x5=21990 | 4862x9=43758 | 5429x6=32574 |  |
| 2973x5=14865 | 3497x6=20982 | 4293x6=25758 | 4523x7=31661 | 4872x9=43848 | 5437x8=43496 |  |
| 2974x5=14870 | 3498x2=6996 | 4325x7=30275 | 4539x7=31773 | 4879x3=14637 | 5473x8=43784 |  |
| 5483x6=32898 | 6342x7=44394 | 6982x7=48874 | 7942x8=63536 | 8635x4=34540 | 9352x7=65464 |  |
| 5492x8=43936 | 6347x8=50776 | 6985x2=13970 | 7943x6=47658 | 8639x4=34556 | 9364x7=65548 |  |
| 5493x8=43944 | 6349x8=50792 | 6985x7=48895 | 7946x8=63568 | 8642x7=60494 | 9372x8=74976 |  |
| 5496x8=43968 | 6354x7=44478 | 7234x8=57872 | 7954x3=23862 | 8642x9=77778 | 9374x5=46870 |  |
| 5497x3=16491 | 6392x7=44744 | 7236x8=57888 | 7958x3=23874 | 8647x3=25941 | 9378x5=46890 |  |
| 5624x7=39368 | 6395x7=44765 | 7243x8=57944 | 7962x8=63696 | 8649x3=25947 | 9385x7=65695 |  |
| 5639x7=39473 | 6397x4=25588 | 7293x6=43758 | 7963x4=31852 | 8653x7=60571 | 9425x6=56550 |  |
| 5679x2=11358 | 6397x5=31985 | 7294x6=43764 | 7968x2=15936 | 8695x2=17390 | 9425x7=65975 |  |
| 5683x7=39781 | 6398x7=44786 | 7345x8=58760 | 7984x3=23952 | 8697x4=34788 | 9428x6=56568 |  |
| 5684x7=39788 | 6423x7=44961 | 7346x8=58768 | 8234x7=57638 | 8734x5=43670 | 9432x8=75456 |  |
| 5689x2=11378 | 6428x7=44996 | 7362x8=58896 | 8239x7=57673 | 8736x4=34944 | 9435x8=75480 |  |
| 5689x4=22756 | 6472x8=51776 | 7365x4=29460 | 8245x6=49470 | 8736x5=43680 | 9437x8=75496 |  |
| 5694x7=39858 | 6475x3=19425 | 7385x4=29540 | 8247x6=49482 | 8739x5=43695 | 9456x3=28368 |  |
| 5698x2=11396 | 6478x3=19434 | 7392x5=36960 | 8253x7=57771 | 8746x3=26238 | 9472x6=56832 |  |
| 5732x8=45856 | 6493x8=51944 | 7398x4=29592 | 8264x7=57848 | 8764x9=78876 | 9473x6=56838 |  |
| 5739x4=22956 | 6495x8=51960 | 7432x6=44592 | 8293x6=49758 | 8765x9=78885 | 9476x3=28428 |  |
| 5743x6=34458 | 6542x9=58878 | 7492x8=59936 | 8295x6=49770 | 8792x6=52752 | 9483x6=56898 |  |
| 5746x3=17238 | 6578x3=19734 | 7498x6=44988 | 8326x7=58282 | 8793x6=52758 | 9486x3=28458 |  |
| 5763x9=51867 | 6587x3=19761 | 7532x9=67788 | 8329x6=49974 | 8795x3=26385 | 9524x7=66668 |  |
| 5798x3=17394 | 6597x3=19791 | 7584x3=22752 | 8356x7=58492 | 8796x5=43980 | 9526x7=66682 |  |
| 5823x6=34938 | 6732x9=60588 | 7594x3=22782 | 8369x7=58583 | 8923x6=53538 | 9542x7=66794 |  |
| 5832x7=40824 | 6739x4=26956 | 7623x8=60984 | 8372x5=41860 | 8923x7=62461 | 9546x3=28638 |  |
| 5842x7=40894 | 6748x3=20244 | 7645x3=22935 | 8392x5=41960 | 8924x7=62468 | 9564x3=28692 |  |
| 5863x4=23452 | 6785x3=20355 | 7652x9=68868 | 8394x7=58758 | 8935x4=35740 | 9564x7=66948 |  |
| 5923x7=41461 | 6793x5=33965 | 7653x9=68877 | 8397x4=33588 | 8936x4=35744 | 9584x3=28752 |  |
| 5927x6=35562 | 6794x3=20382 | 7658x3=22974 | 8397x5=41985 | 8937x5=44685 | 9586x3=28758 |  |
| 5928x7=41496 | 6795x3=20385 | 7685x4=30740 | 8423x7=58961 | 8953x7=62671 | 9587x3=28761 |  |
| 5932x6=35592 | 6835x4=27340 | 7689x4=30756 | 8425x6=50550 | 8954x3=26862 | 9623x7=67361 |  |
| 5937x8=47496 | 6853x9=61677 | 7839x4=31356 | 8429x6=50574 | 8954x7=62678 | 9623x8=76984 |  |
| 5942x7=41594 | 6859x3=20577 | 7846x3=23538 | 8432x6=50592 | 8956x7=62692 | 9632x7=67424 |  |
| 5943x6=35658 | 6875x2=13750 | 7856x3=23568 | 8457x3=25371 | 8962x7=62734 | 9638x4=38552 |  |
| 5943x8=47544 | 6897x4=27588 | 7859x3=23577 | 8479x3=25437 | 8963x7=62741 | 9645x3=28935 |  |
| 5948x6=35688 | 6925x7=48475 | 7863x9=70767 | 8495x6=50970 | 8965x3=26895 | 9675x2=19350 |  |
| 5963x4=23852 | 6934x5=34670 | 7864x3=23592 | 8496x3=25488 | 8974x3=26922 | 9682x7=67774 |  |
| 5968x4=23872 | 6934x8=55472 | 7865x4=31460 | 8523x7=59661 | 8976x5=44880 | 9685x7=67795 |  |
| 5983x6=35898 | 6972x8=55776 | 7924x6=47544 | 8536x7=59752 | 9235x8=73880 | 9724x6=58344 |  |
| 5986x3=17958 | 6973x4=27892 | 7925x6=47550 | 8549x3=25647 | 9247x8=73976 | 9725x6=58350 |  |
| 6238x7=43666 | 6973x8=55784 | 7928x6=47568 | 8564x3=25692 | 9254x7=64778 | 9728x6=58368 |  |
| 6239x7=43673 | 6974x3=20922 | 7934x8=63472 | 8564x7=59948 | 9285x7=64995 | 9743x6=58458 |  |
| 6243x8=49944 | 6975x3=20925 | 7936x4=31744 | 8576x3=25728 | 9342x7=65394 | 9743x8=77944 |  |
| 6249x8=49992 | 6978x2=13956 | 7936x5=39680 | 8623x7=60361 | 9342x8=74736 | 9745x6=58470 |  |
| 9756x3=29268 | 9827x6=58962 | 9834x7=68838 | 9842x7=68894 | 9865x4=39460 |  |  |
| 9825x7=68775 | 9832x7=68824 | 9836x4=39344 | 9847x3=29541 | 9867x4=39468 |  |  |

| **Type 2x2 problem** | | | | | | |
| --- | --- | --- | --- | --- | --- | --- |
| 23x65=1495 | 28x34=952 | 36x85=3060 | 43x65=2795 | 52x89=4628 | 58x37=2146 |  |
| 23x76=1748 | 28x43=1204 | 36x94=3384 | 43x68=2924 | 52x96=4992 | 58x43=2494 |  |
| 23x79=1817 | 28x59=1652 | 36x95=3420 | 43x76=3268 | 53x47=2491 | 58x46=2668 |  |
| 23x85=1955 | 28x76=2128 | 37x48=1776 | 43x95=4085 | 53x48=2544 | 58x47=2726 |  |
| 23x86=1978 | 29x34=986 | 37x49=1813 | 43x97=4171 | 53x49=2597 | 58x63=3654 |  |
| 23x96=2208 | 29x43=1247 | 37x64=2368 | 43x98=4214 | 53x68=3604 | 58x67=3886 |  |
| 23x98=2254 | 29x56=1624 | 37x68=2516 | 45x37=1665 | 53x69=3657 | 58x73=4234 |  |
| 24x65=1560 | 29x67=1943 | 37x86=3182 | 45x38=1710 | 53x78=4134 | 58x74=4292 |  |
| 24x68=1632 | 29x78=2262 | 37x89=3293 | 45x69=3105 | 53x96=5088 | 58x76=4408 |  |
| 24x69=1656 | 32x57=1824 | 37x94=3478 | 45x87=3915 | 54x96=5184 | 59x27=1593 |  |
| 24x75=1800 | 32x58=1856 | 37x96=3552 | 45x96=4320 | 56x23=1288 | 59x34=2006 |  |
| 24x79=1896 | 32x69=2208 | 38x46=1748 | 45x97=4365 | 56x24=1344 | 59x37=2183 |  |
| 24x85=2040 | 32x76=2432 | 38x54=2052 | 45x98=4410 | 56x29=1624 | 59x46=2714 |  |
| 24x86=2064 | 32x89=2848 | 38x56=2128 | 46x35=1610 | 56x32=1792 | 59x63=3717 |  |
| 24x95=2280 | 32x97=3104 | 38x64=2432 | 46x38=1748 | 56x37=2072 | 59x84=4956 |  |
| 24x96=2304 | 32x98=3136 | 38x65=2470 | 46x85=3910 | 56x38=2128 | 62x58=3596 |  |
| 25x34=850 | 34x25=850 | 38x74=2812 | 46x87=4002 | 56x43=2408 | 63x47=2961 |  |
| 25x43=1075 | 34x26=884 | 38x79=3002 | 46x89=4094 | 56x49=2744 | 63x49=3087 |  |
| 25x67=1675 | 34x27=918 | 39x45=1755 | 46x95=4370 | 56x72=4032 | 63x58=3654 |  |
| 25x68=1700 | 34x28=952 | 39x68=2652 | 46x97=4462 | 56x74=4144 | 63x59=3717 |  |
| 25x76=1900 | 34x29=986 | 39x85=3315 | 47x38=1786 | 56x83=4648 | 63x84=5292 |  |
| 25x78=1950 | 34x56=1904 | 42x56=2352 | 47x56=2632 | 56x89=4984 | 63x87=5481 |  |
| 25x86=2150 | 34x85=2890 | 42x57=2394 | 48x56=2688 | 56x92=5152 | 63x95=5985 |  |
| 25x89=2225 | 34x87=2958 | 42x59=2478 | 48x57=2736 | 57x29=1653 | 64x38=2432 |  |
| 25x97=2425 | 34x98=3332 | 42x65=2730 | 48x69=3312 | 57x32=1824 | 64x39=2496 |  |
| 26x34=884 | 35x47=1645 | 42x67=2814 | 48x79=3792 | 57x34=1938 | 64x53=3392 |  |
| 26x43=1118 | 35x49=1715 | 42x78=3276 | 49x35=1715 | 57x36=2052 | 64x58=3712 |  |
| 26x58=1508 | 35x64=2240 | 42x79=3318 | 49x53=2597 | 57x39=2223 | 64x59=3776 |  |
| 26x59=1534 | 35x69=2415 | 42x85=3570 | 49x56=2744 | 57x43=2451 | 64x79=5056 |  |
| 26x78=2028 | 35x74=2590 | 42x86=3612 | 49x58=2842 | 57x46=2622 | 64x83=5312 |  |
| 26x87=2262 | 35x76=2660 | 42x87=3654 | 49x63=3087 | 57x63=3591 | 64x85=5440 |  |
| 26x97=2522 | 35x78=2730 | 42x89=3738 | 49x65=3185 | 57x82=4674 | 64x89=5696 |  |
| 26x98=2548 | 35x86=3010 | 43x25=1075 | 49x73=3577 | 57x92=5244 | 64x97=6208 |  |
| 27x34=918 | 35x89=3115 | 43x26=1118 | 49x76=3724 | 57x96=5472 | 64x98=6272 |  |
| 27x43=1161 | 35x96=3360 | 43x27=1161 | 52x68=3536 | 58x26=1508 | 65x23=1495 |  |
| 27x65=1755 | 35x98=3430 | 43x28=1204 | 52x78=4056 | 58x29=1682 | 65x24=1560 |  |
| 27x69=1863 | 36x78=2808 | 43x29=1247 | 52x79=4108 | 58x34=1972 | 65x39=2535 |  |
| 65x43=2795 | 69x75=5175 | 76x82=6232 | 84x59=4956 | 89x34=3026 | 96x23=2208 |  |
| 65x47=3055 | 69x83=5727 | 76x85=6460 | 84x67=5628 | 89x35=3115 | 96x25=2400 |  |
| 65x49=3185 | 69x84=5796 | 76x94=7144 | 84x76=6384 | 89x37=3293 | 96x27=2592 |  |
| 65x72=4680 | 72x59=4248 | 76x95=7220 | 84x93=7812 | 89x42=3738 | 96x28=2688 |  |
| 65x79=5135 | 72x96=6912 | 78x34=2652 | 84x95=7980 | 89x45=4005 | 96x32=3072 |  |
| 65x83=5395 | 72x98=7056 | 78x36=2808 | 84x97=8148 | 89x62=5518 | 96x42=4032 |  |
| 65x94=6110 | 73x56=4088 | 78x39=3042 | 85x23=1955 | 89x72=6408 | 96x45=4320 |  |
| 67x23=1541 | 73x69=5037 | 78x42=3276 | 85x43=3655 | 89x73=6497 | 96x52=4992 |  |
| 67x24=1608 | 73x85=6205 | 78x43=3354 | 85x46=3910 | 92x56=5152 | 96x57=5472 |  |
| 67x29=1943 | 73x86=6278 | 78x45=3510 | 85x47=3995 | 92x57=5244 | 96x74=7104 |  |
| 67x34=2278 | 73x94=6862 | 79x23=1817 | 85x62=5270 | 92x58=5336 | 96x82=7872 |  |
| 67x35=2345 | 74x53=3922 | 79x24=1896 | 85x63=5355 | 92x65=5980 | 96x85=8160 |  |
| 67x38=2546 | 74x56=4144 | 79x25=1975 | 85x67=5695 | 93x64=5952 | 97x23=2231 |  |
| 67x42=2814 | 74x59=4366 | 79x34=2686 | 85x74=6290 | 93x78=7254 | 97x25=2425 |  |
| 67x48=3216 | 74x65=4810 | 79x35=2765 | 85x92=7820 | 93x86=7998 | 97x26=2522 |  |
| 67x53=3551 | 74x68=5032 | 79x45=3555 | 86x24=2064 | 93x87=8091 | 97x28=2716 |  |
| 67x82=5494 | 74x89=6586 | 79x56=4424 | 86x27=2322 | 94x36=3384 | 97x32=3104 |  |
| 67x83=5561 | 75x23=1725 | 79x65=5135 | 86x29=2494 | 94x37=3478 | 97x35=3395 |  |
| 67x84=5628 | 75x24=1800 | 82x57=4674 | 86x32=2752 | 94x63=5922 | 97x38=3686 |  |
| 67x85=5695 | 75x38=2850 | 82x59=4838 | 86x37=3182 | 94x68=6392 | 97x53=5141 |  |
| 67x93=6231 | 75x42=3150 | 82x67=5494 | 86x43=3698 | 94x75=7050 | 97x56=5432 |  |
| 67x95=6365 | 75x49=3675 | 82x75=6150 | 86x45=3870 | 94x76=7144 | 97x63=6111 |  |
| 68x23=1564 | 75x69=5175 | 82x76=6232 | 86x47=4042 | 94x87=8178 | 97x83=8051 |  |
| 68x25=1700 | 75x83=6225 | 82x95=7790 | 86x75=6450 | 95x24=2280 | 98x23=2254 |  |
| 68x39=2652 | 75x84=6300 | 83x45=3735 | 86x95=8170 | 95x26=2470 | 98x24=2352 |  |
| 68x45=3060 | 75x86=6450 | 83x49=4067 | 87x23=2001 | 95x27=2565 | 98x27=2646 |  |
| 68x52=3536 | 75x93=6975 | 83x54=4482 | 87x24=2088 | 95x34=3230 | 98x34=3332 |  |
| 68x57=3876 | 75x94=7050 | 83x59=4897 | 87x29=2523 | 95x36=3420 | 98x36=3528 |  |
| 68x72=4896 | 75x96=7200 | 83x64=5312 | 87x34=2958 | 95x42=3990 | 98x45=4410 |  |
| 68x74=5032 | 76x28=2128 | 83x74=6142 | 87x42=3654 | 95x46=4370 | 98x46=4508 |  |
| 69x23=1587 | 76x32=2432 | 83x76=6308 | 87x43=3741 | 95x47=4465 | 98x52=5096 |  |
| 69x37=2553 | 76x34=2584 | 83x79=6557 | 87x45=3915 | 95x48=4560 | 98x64=6272 |  |
| 69x38=2622 | 76x35=2660 | 83x94=7802 | 87x46=4002 | 95x62=5890 | 98x74=7252 |  |
| 69x52=3588 | 76x39=2964 | 83x96=7968 | 87x53=4611 | 95x63=5985 |  |  |
| 69x54=3726 | 76x45=3420 | 84x35=2940 | 87x65=5655 | 95x73=6935 |  |  |
| 69x72=4968 | 76x53=4028 | 84x39=3276 | 87x92=8004 | 95x83=7885 |  |  |
| 69x73=5037 | 76x58=4408 | 84x57=4788 | 89x24=2136 | 95x86=8170 |  |  |

**Appendix 2**

**In Experiment 2: Both types of multidigit multiplication problems and their correct answers.**

*Note.* In Experiment 2, participants were assigned 100 problems in total, including type 2x1 and 4x1 conditions, in which were 50 problems respectively. The problems of each condition were randomly selected from this item pool by matlab (2008b) program. The total 100 problems every participant calculated in the experiment were not necessarily the same.

| **Type 2x1 problem** | | | **Type 4x1 problem** | | |
| --- | --- | --- | --- | --- | --- |
| 23x4=92 | 37x9=333 | 62x7=434 | 2369x7=16583 | 3749x6=22494 | 6237x8=49896 |
| 23x6=138 | 38x2=76 | 62x8=496 | 2384x7=16688 | 3782x6=22692 | 6372x8=50976 |
| 23x7=161 | 38x4=152 | 62x9=558 | 2478x6=14868 | 3794x6=22764 | 6387x4=25548 |
| 23x8=184 | 38x6=228 | 63x4=252 | 2497x8=19976 | 3798x6=22788 | 6497x8=51976 |
| 23x9=207 | 38x7=266 | 63x7=441 | 2638x7=18466 | 3824x6=22944 | 6498x3=19494 |
| 24x3=72 | 38x9=342 | 63x8=504 | 2748x6=16488 | 3824x7=26768 | 6732x9=60588 |
| 24x6=144 | 39x2=78 | 63x9=567 | 2839x7=19873 | 3874x9=34866 | 6823x7=47761 |
| 24x7=168 | 39x4=156 | 64x3=192 | 2864x9=25776 | 3924x7=27468 | 6839x7=47873 |
| 24x8=192 | 39x6=234 | 64x7=448 | 2938x7=20566 | 3947x8=31576 | 6849x3=20547 |
| 24x9=216 | 39x7=273 | 64x8=512 | 2973x6=17838 | 3968x4=15872 | 6872x9=61848 |
| 26x3=78 | 39x8=312 | 64x9=576 | 2983x7=20881 | 3972x8=31776 | 6874x3=20622 |
| 26x7=182 | 42x6=252 | 67x2=134 | 3298x6=19788 | 3984x7=27888 | 6897x4=27588 |
| 26x8=208 | 42x7=294 | 67x3=201 | 3426x7=23982 | 4237x8=33896 | 6947x3=20841 |
| 26x9=234 | 42x8=336 | 67x4=268 | 3428x6=20568 | 4273x6=25638 | 6947x8=55576 |
| 27x3=81 | 42x9=378 | 67x8=536 | 3429x6=20574 | 4293x6=25758 | 6973x4=27892 |
| 27x6=162 | 43x6=258 | 67x9=603 | 3467x2=6934 | 4297x6=25782 | 6973x8=55784 |
| 27x9=243 | 43x7=301 | 68x2=136 | 3468x2=6936 | 4367x2=8734 | 6974x3=20922 |
| 28x3=84 | 43x8=344 | 68x3=204 | 3469x2=6938 | 4368x2=8736 | 6987x2=13974 |
| 28x6=168 | 43x9=387 | 68x4=272 | 3472x6=20832 | 4369x2=8738 | 7243x8=57944 |
| 28x7=196 | 46x2=92 | 68x7=476 | 3476x2=6952 | 4372x8=34976 | 7249x6=43494 |
| 28x9=252 | 46x3=138 | 68x9=612 | 3478x2=6956 | 4376x2=8752 | 7293x6=43758 |
| 29x3=87 | 46x7=322 | 69x2=138 | 3479x2=6958 | 4378x2=8756 | 7294x6=43764 |
| 29x6=174 | 46x8=368 | 69x3=207 | 3486x2=6972 | 4379x2=8758 | 7298x6=43788 |
| 29x7=203 | 46x9=414 | 69x4=276 | 3487x2=6974 | 4386x2=8772 | 7398x4=29592 |
| 29x8=232 | 47x2=94 | 69x7=483 | 3489x2=6978 | 4387x2=8774 | 7428x6=44568 |
| 32x6=192 | 47x3=141 | 69x8=552 | 3496x2=6992 | 4389x2=8778 | 7492x6=44952 |
| 32x7=224 | 47x6=282 | 72x6=432 | 3496x2=6992 | 4396x2=8792 | 7492x8=59936 |
| 34x6=204 | 47x8=376 | 72x8=576 | 3497x2=6994 | 4397x2=8794 | 7493x8=59944 |
| 34x7=238 | 47x9=423 | 72x9=648 | 3497x6=20982 | 4398x2=8796 | 7496x3=22488 |
| 34x9=306 | 48x2=96 | 73x4=292 | 3497x8=27976 | 4398x7=30786 | 7496x8=59968 |
| 36x2=72 | 48x3=144 | 73x6=438 | 3498x2=6996 | 4628x7=32396 | 7639x4=30556 |
| 36x4=144 | 48x6=288 | 73x9=657 | 3624x7=25368 | 4723x8=37784 | 7832x6=46992 |
| 36x7=252 | 48x7=336 | 74x3=222 | 3624x8=28992 | 4872x9=43848 | 7836x4=31344 |
| 36x8=288 | 48x9=432 | 74x8=592 | 3628x7=25396 | 4876x3=14628 | 7842x9=70578 |
| 36x9=324 | 49x2=98 | 74x9=666 | 3642x9=32778 | 4876x9=43884 | 7864x9=70776 |
| 37x2=74 | 49x3=147 | 76x2=152 | 3687x4=14748 | 4972x6=29832 | 7924x6=47544 |
| 37x4=148 | 49x6=294 | 76x3=228 | 3689x4=14756 | 4978x3=14934 | 7936x4=31744 |
| 37x6=222 | 49x7=343 | 76x8=608 | 3729x6=22374 | 4982x6=29892 | 7938x4=31752 |
| 37x8=296 | 49x8=392 | 76x9=684 | 3742x6=22452 | 4986x3=14958 | 7943x6=47658 |
| 78x2=156 | 86x4=344 | 94x7=658 | 7946x3=23838 | 8932x7=62524 | 9724x8=77792 |
| 78x3=234 | 86x7=602 | 94x8=752 | 7948x6=47688 | 8937x4=35748 | 9732x8=77856 |
| 78x4=312 | 86x9=774 | 96x2=192 | 7962x8=63696 | 8963x7=62741 | 9734x8=77872 |
| 78x6=468 | 87x2=174 | 96x3=288 | 8264x7=57848 | 8967x2=17934 | 9742x6=58452 |
| 78x9=702 | 87x3=261 | 96x4=384 | 8273x6=49638 | 9234x7=64638 | 9743x6=58458 |
| 79x2=158 | 87x4=348 | 96x7=672 | 8293x6=49758 | 9243x6=55458 | 9746x8=77968 |
| 79x3=237 | 87x6=522 | 97x2=194 | 8326x7=58282 | 9264x7=64848 | 9784x3=29352 |
| 79x4=316 | 87x9=783 | 97x3=291 | 8369x4=33476 | 9268x7=64876 | 9786x2=19572 |
| 79x6=474 | 89x2=178 | 97x6=582 | 8369x7=58583 | 9273x6=55638 | 9827x6=58962 |
| 79x8=632 | 89x3=267 | 97x8=776 | 8392x7=58744 | 9283x7=64981 | 9832x6=58992 |
| 82x6=492 | 89x4=356 | 98x2=196 | 8394x7=58758 | 9342x7=65394 | 9842x7=68894 |
| 83x4=332 | 89x6=534 | 98x3=294 | 8432x6=50592 | 9347x8=74776 | 9846x3=29538 |
| 83x6=498 | 89x7=623 | 98x4=392 | 8632x9=77688 | 9367x4=37468 | 9847x3=29541 |
| 83x7=581 | 92x7=644 | 98x6=588 | 8679x2=17358 | 9423x7=65961 | 9867x2=19734 |
| 83x9=747 | 92x8=736 | 98x7=686 | 8694x7=60858 | 9427x6=56562 | 9876x2=19752 |
| 84x3=252 | 93x4=372 |  | 8739x4=34956 | 9432x6=56592 |  |
| 84x6=504 | 93x6=558 |  | 8762x9=78858 | 9432x8=75456 |  |
| 84x7=588 | 93x7=651 |  | 8763x9=78867 | 9436x8=75488 |  |
| 84x9=756 | 93x8=744 |  | 8796x3=26388 | 9437x8=75496 |  |
| 86x2=172 | 94x3=282 |  | 8926x7=62482 | 9483x6=56898 |  |
| 86x3=258 | 94x6=564 |  | 8927x6=53562 | 9683x4=38732 |  |
